# T1WI‐SWI Dual Modal Magnetic Resonance Nanoprobes for Accurate Diagnosis of Early Stage Alzheimer's Disease

**DOI:** 10.1002/advs.202510298

**Published:** 2025-10-27

**Authors:** Minghua Li, Aijun Shen, Xiaolong Gao, Chao Lin, Zongliang Huang, Qi Lv, Junjun Tang, Xiaolong Ma, Jiong Ni, Ju Tian, Jiaqi Wu, Xiaowen Xu, Wei Wang, Peijun Wang

**Affiliations:** ^1^ Department of Medical Imaging Tongji Hospital School of Medicine Tongji University Shanghai 200092 P. R. China; ^2^ Institute of Medical Imaging Artificial Intelligence Tongji University School of Medicine Shanghai 200092 P. R. China; ^3^ Department of Radiology Luodian Hospital Shanghai University Shanghai 201908 P. R. China; ^4^ Department of Radiology Luodian Hospital Baoshan Shanghai 201908 P. R. China; ^5^ Department of Periodontology School and Hospital of Stomatology Shanghai Engineering Research Center of Tooth Restoration and Regeneration Tongji University Shanghai 200072 P. R China; ^6^ School of Medicine Tongji University Shanghai 200092 P. R. China

**Keywords:** alzheimer's disease, magnetic resonance imaging, magnetic resonance tuning, susceptibility weighted imaging

## Abstract

Beta‐site APP‐cleaving enzyme 1 (BACE1), a critical rate‐limiting enzyme that synthesizes β‐amyloid peptide (Aβ), is an important marker of early pathological changes in Alzheimer's disease (AD). Early small plaques cannot be accurately detected using traditional Magnetic resonance imaging (MRI) probes. Therefore, magnetic resonance tuning (MRET) and susceptibility weighted imaging (SWI)‐based smart responsive MR nanoprobes are designed to achieve the sensitive detection of BACE1 and Aβ plaques. This probe is modified with a blood‐brain barrier‐penetrating targeting peptide that enables its reach to the AD microenvironment. The enhancement of T1WI signals owing to the MRET effect caused by the separation of probes in response to BACE1 is used to reflect real‐time BACE1 changes. When Aβ plaques are present, the remaining probes that bound around Aβ plaques underwent in situ thiol cross‐linking under the action of peroxynitrite (ONOO^−^) in the AD microenvironment, and SWI can magnify magnetic susceptibility differences, which significantly increased the Aβ plaque detection sensitivity. This probe combines the advantages of T1WI and SWI and can simultaneously visualize and reflect the BACE1 level and Aβ plaque distribution. This approach overcomes the limitations of traditional single‐target probes and provides a new noninvasive and highly accurate strategy for the early diagnosis of AD.

## Graphical abstract

We developed a smart MRI nanoprobe targeting BACE1 and Aβ plaques. It crosses the blood‐brain barrier. Upon encountering BACE1, the probe is cleaved, enabling T1WI imaging detection. Subsequent reaction with ONOO^−^ induces probe aggregation, enhancing magnetic susceptibility for sensitive SWI imaging of Aβ plaques. This dual‐modal probe allows early AD diagnosis before spatial learning memory impairment, enabling pathological progression monitoring.

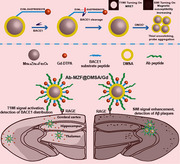

## 1 Introduction

Alzheimer's disease (AD), characterized by progressive cognitive decline and neuropathological hallmarks including β‐amyloid (Aβ) plaques and tau tangles,^[^ ^1^, ^2^, ^3^ ^]^ lacks reliable early specific diagnostic markers.^[^ ^4^ ^]^ Pathological changes in AD, known as “preclinical AD”, start to occur 10–20 years before clinical symptoms.^[^ ^5^ ^]^ Therefore, developing a non‐invasive diagnostic technology to detect early molecular events is key for early discovery, diagnosis, and intervention to slow disease progression as well as improve the survival rates of patients with AD.

Beta‐site APP‐cleaving enzyme 1 (BACE1), the rate‐limiting enzyme in Aβ generation,^[^ ^6^ ^]^ is a critical biomarker for early AD pathology, preceding Aβ deposition^[^ ^7^, ^8^ ^]^ and correlating with progression from mild cognitive impairment (MCI) to AD.^[^ ^9^ ^]^ Many clinical trials of AD treatment have shown that interventions at the MCI stage are usually effective.^[^ ^10^ ^]^ BACE1 detection is vital for the early treatment of AD. However, current BACE1 detection methods rely on fluorescent probes with limited clinical applicability.^[^ ^11^, ^12^, ^13^ ^]^ Thus, developing a clinically translatable, non‐invasive, and highly sensitive in situ imaging method for brain BACE1 is essential.

**Scheme 1:**
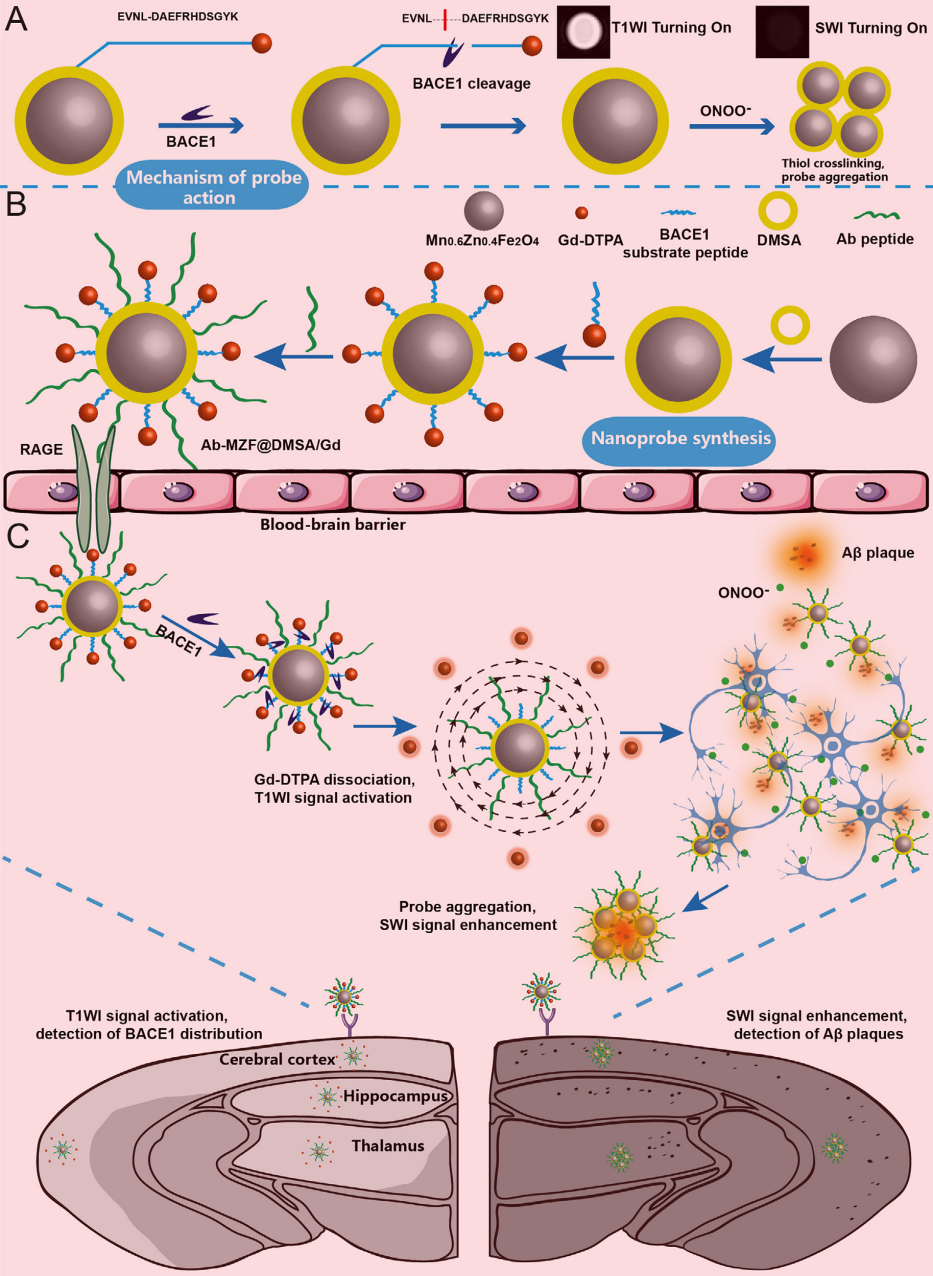
A) Schematic illustration of the mechanism of Ab‐MZF@DMSA/Gd, showing BACE1 enzyme‐activated MRET effect for turn‐on T1WI imaging and ONOO^−^‐induced probe aggregation to enhance susceptibility for SWI signal amplification. B) Schematic illustration of the construction of the smart responsive MR nanoprobe Ab‐MZF@DMSA/Gd. C) Schematic illustration of Ab‐MZF@DMSA/Gd for precise detection and visualization of BACE1 and Aβ plaques in early‐stage AD brain.

Magnetic resonance imaging (MRI) offers a high soft tissue resolution and has clinical utility. Distance‐dependent magnetic resonance tuning (MRET) enables T1WI signal switching via the spatial separation of paramagnetic enhancers and superparamagnetic quenchers,^[^ ^14^, ^15^, ^16^, ^17^ ^]^ providing a potential platform for BACE1 visualization (**Scheme** **1**A). Simultaneously, Aβ plaques occur several years before cognitive impairment occurs in AD patients,^[^ ^18^, ^19^ ^]^ but the only existing T1‐ or T2‐targeting contrast agents suffer from low sensitivity and high background noise, hindering accurate early detection.^[^ ^20^, ^21^, ^22^ ^]^

Peroxynitrite (ONOO^−^) is a key mediator in AD neuroinflammation^[^ ^23^ ^]^ and Aβ aggregation.^[^ ^24^ ^]^ ONOO^−^ and Aβ aggregation are two critical events that mutually amplify each other through positive feedback and jointly promote AD occurrence and progression. Susceptibility‐weighted imaging (SWI), a functional imaging technique based on differences in tissue magnetic susceptibility, is highly sensitive to changes in magnetic susceptibility and is 3–6 times more sensitive than routine MRI techniques.^[^ ^25^ ^]^ We designed a smart SWI molecular probe that responds to ONOO^−^. High‐sensitivity detection of Aβ plaques was achieved by enhancing magnetic susceptibility toward amplification of the SWI signal via probe self‐aggregation under the effects of ONOO^−^ (Scheme 1A).

Thus, to monitor BACE1 in the brains of patients with AD and accurately and sensitively display small Aβ plaques in early‐stage AD, we designed a smart responsive MR nanoprobe based on MRET and SWI. In previous studies, we prepared superparamagnetic Mn, Zn‐doped ferrite magnetic nanoparticles (MZF‐MNPs)—Mn_0.6_Zn_0.4_Fe_2_O_4_ with higher magnetization than typical Fe_3_O_4_‐SPIO particles,^[^ ^26^, ^27^ ^]^ to achieve more effective quenching. In this study, we designed nanoprobes with a Mn_0.6_Zn_0.4_Fe_2_O_4_ core. First, dimercaptosuccinic acid (DMSA)‐modified Mn_0.6_Zn_0.4_Fe_2_O_4_ was used to synthesize ONOO^−^‐responsive nanoprobes (MZF@DMSA). Next, the BACE1 smart response and efficient MRET were combined to construct a smart MR molecular probe that could accurately measure brain BACE1 levels (MZF@DMSA/Gd). This probe comprises a superparamagnetic MZF@DMSA (T1 quencher) and a paramagnetic copolymer Gd‐DTPA (T1 enhancer). The two molecules are connected by a BACE1 substrate (EVNL‐DAEFRHDSGYK).^[^ ^28^, ^29^ ^]^ Finally, the nanoprobe surface was functionalized with KLVFFAED peptide (Ab peptide) as a receptor for advanced glycation end products (RAGE)‐mediated blood‐brain barrier (BBB) penetration and active Aβ plaques targeting,^[^ ^30^, ^31^ ^]^ ultimately yielding a smart responsive MR nanoprobe (Ab‐MZF@DMSA/Gd) for early AD diagnosis (Scheme 1B). After the probe penetrates the BBB and enters the brain, high BACE1 levels in the AD brain cleave the peptide substrate, causing the T1 quencher MZF@DMSA and the T1 enhancer Gd‐DTPA to separate. Owing to the MRET effect, T1WI is switched on, achieving real‐time detection of BACE1 changes in the brain (Scheme 1C). If Aβ plaques are already present in the brain, the remaining nanoprobes will target the Aβ plaques. Under the action of ONOO^−^, in situ cross‐linking of the thiol groups in MZF@DMSA occurs, causing probe aggregation. This increases magnetic susceptibility and enhances SWI signals, thereby accurately and sensitively revealing Aβ plaques (Scheme 1C). We used T1WI and SWI dual imaging for visualizable quantification and visualization of BACE1 and Aβ plaques in the brain, thereby allowing accurate evaluation of early pathological changes in AD. This approach enables the early diagnosis and simultaneous monitoring of the pathological progression of AD.

## 2 Results and Discussion

### 2.1 Synthesis and Characterization of Ab‐MZF@DMSA/Gd

**Figure 1:**
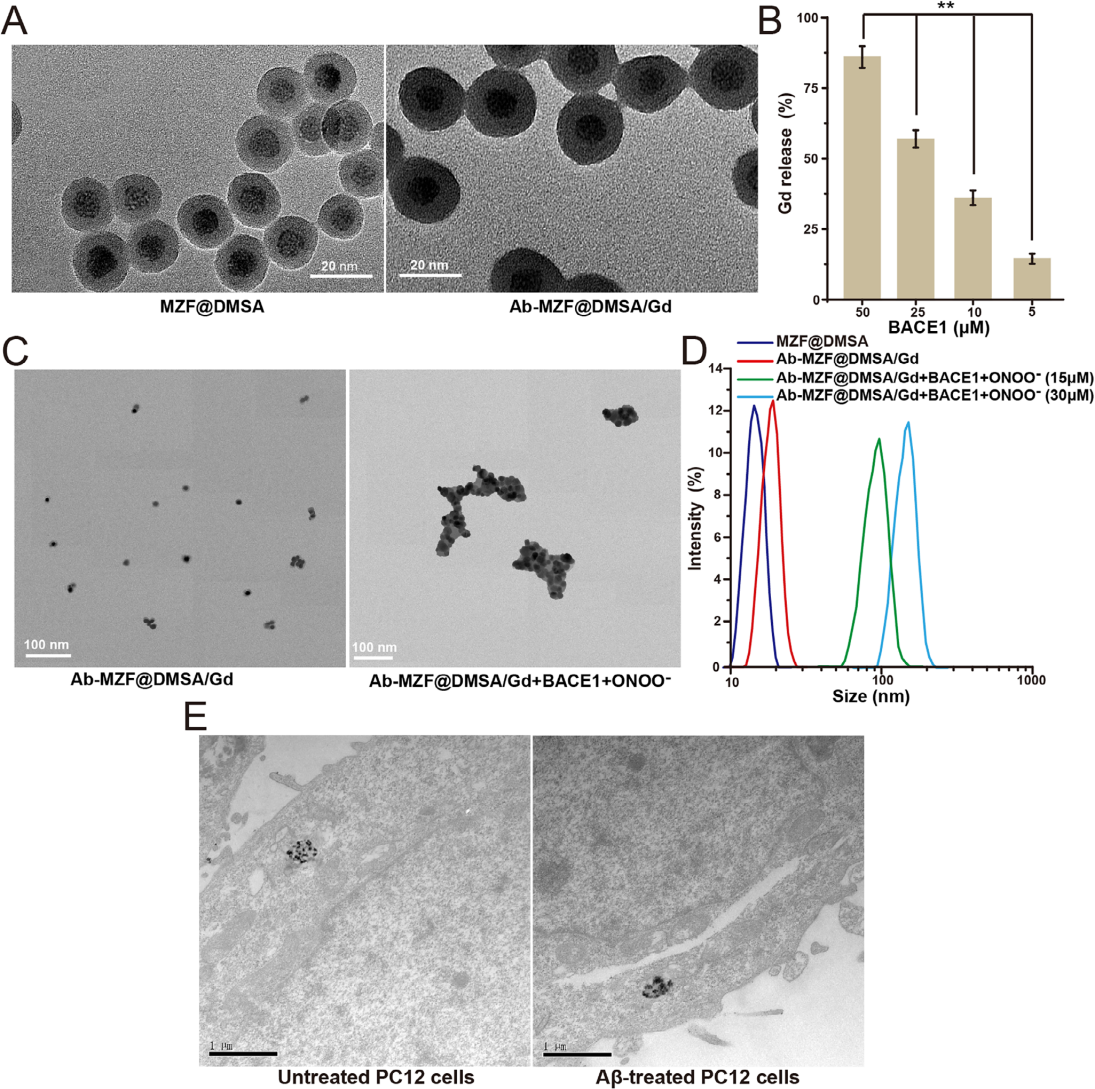
A) TEM images of MZF@DMSA and Ab‐MZF@DMSA/Gd. B) Gadolinium ions release ratios of Ab‐MZF@DMSA/Gd after adding various concentrations of BACE1 (n = 3). C) TEM images of Ab‐MZF@DMSA/Gd and Ab‐MZF@DMSA/Gd+BACE1 treated with ONOO^−^. D) DLS measurements of MZF@DMSA, Ab‐MZF@DMSA/Gd, and Ab‐MZF@DMSA/Gd+BACE1 treated with various concentrations of ONOO^−^ (n = 3). E) TEM images of Aβ‐treated and untreated PC12 cells after incubation with Ab‐MZF@DMSA/Gd+BACE1. Statistical significance is indicated (^**^ *p* < 0.01) by Student's *t*‐test.

The Mn_0.6_Zn_0.4_Fe_2_O_4_ nanoparticles prepared via liquid‐phase thermal decomposition exhibited good monodispersity and a diameter of 8 nm (Figure S1A, Supporting Information). X‐ray diffraction (XRD) revealed that Mn_0.6_Zn_0.4_Fe_2_O_4_ has a single‐phase spinel structure (Figure S1B, Supporting Information). To produce nanoparticles responsive to ONOO^−^, we used DMSA to coat them with Mn_0.6_Zn_0.4_Fe_2_O_4_ to obtain MZF@DMSA. Transmission electron microscopy (TEM) displayed MZF@DMSA to have good dispersity and a diameter of 15 nm (**Figure** **1**A).

The DMSA on the surface of MZF@DMSA allows the nanoparticles to remain stable under physiological conditions and simultaneously provides two functional groups (carboxyl and thiol).^[^ ^32^ ^]^ Owing to the asymmetric and symmetric vibrations of the carboxyl groups in DMSA, two broad bands at 1685 and 1420 cm^−1^ are present in the infrared spectrum of DMSA.^[^ ^33^ ^]^ A comparison of the infrared spectra of MZF@DMSA and Mn_0.6_Zn_0.4_Fe_2_O_4_ showed two broad bands at 1552 and 1358 cm^−1^ in the infrared spectrum of MZF@DMSA (Figure S2A, Supporting Information), indicating that carboxyl groups were present on the surface of MZF@DMSA and that a partial blue shift was present in the asymmetric and symmetric vibrations of the carboxyl groups.

A sharp characteristic peak was present at 480 cm^−1^ in the Raman spectrum of MZF@DMSA (Figure S2B, Supporting Information), and a partial displacement compared with the Raman characteristic peak (500 cm^−1^) of the S–S stretching vibration has been reported.^[^ ^34^ ^]^ Interestingly, after treatment with 100 µM ONOO^−^, the 480 cm^−1^ Raman peak intensity of MZF@DMSA increased (Figure S2B, Supporting Information). This finding indicates that some disulfide bonds are present in MZF@DMSA. Moreover, some thiol groups may have been present. Under the action of ONOO^−^, some thiol groups in MZF@DMSA formed disulfide bonds, which increased the number of disulfide bonds in MZF@DMSA.

The BACE1 substrate EVNL‐DAEFRHDSGYK was subsequently used to link the T1 quencher, MZF@DMSA, to the T1 enhancer, Gd‐DTPA. BACE1 can cleave the β site with high selectivity, and the peptide substrate is cleaved at the leucine and aspartic acid residues (Scheme 1A). Finally, the probe surface was modified with an Ab peptide (KLVFFAED) that can penetrate the BBB and actively target AD lesions, ultimately yielding a smart‐responsive MR nanoprobe, Ab‐MZF@DMSA/Gd, which can be used for early AD diagnosis. TEM showed that the diameter of the Ab‐MZF@DMSA/Gd particles was 20 nm (Figure 1A).

### 2.2 Responsiveness of Ab‐MZF@DMSA/Gd

We evaluated the BACE1 responsiveness of Ab‐MZF@DMSA/Gd as a BACE1 probe. The BACE1‐treated Ab‐MZF@DMSA/Gd solution was centrifuged, and an inductively coupled plasma optical emission spectrometer (ICP‐OES) was used to measure the gadolinium ion concentration in the supernatant with changes in BACE1 concentration, and the release rate was calculated. After BACE1 was added to Ab‐MZF@DMSA/Gd, the release rate of gadolinium in the supernatant gradually increased as the BACE1 concentration increased (Figure 1B). In contrast, Ab‐MZF@DMSA/Gd is extremely stable in the presence of other proteases (caspase‐3, gamma‐glutamyltranspeptidase (GGT), glutathione (GSH), matrix metalloproteinase‐2 (MMP‐2), acetylcholinesterase (AChE), and butyrylcholinesterase (BChE)), and almost no gadolinium ion release was detected (Figure S3A, Supporting Information). These results revealed that BACE1 could lyse the linker peptide EVNL‐DAEFRHDSGYK in the Ab‐MZF@DMSA/Gd MRET probe in a targeted manner, thereby separating and releasing Gd‐DTPA.

TEM (Figure 1C) displayed that BACE1‐treated Ab‐MZF@DMSA/Gd significantly aggregated in the presence of ONOO^−^. When the ONOO^−^ concentration increased from 0 to 30 µM, the mean hydrated particle size increased from 15 to 160 nm (Figure 1D). We examined the particle size of Ab‐MZF@DMSA/Gd in different metal ion buffers after BACE1 treatment and found that the metal ions did not affect the stability of Ab‐MZF@DMSA/Gd in the respective buffer solutions (Figure S3B, Supporting Information). Moreover, Ab‐MZF@DMSA/Gd was incubated with different reactive oxygen species (ROS) after BACE1 treatment, and there was no significant change in particle size (Figure S3B, Supporting Information). This indicates that the reaction between Ab‐MZF@DMSA/Gd and ONOO^−^ has high specificity. This occurred because carboxyl groups, thild groups, and some disulfide bonds are present on the surface of Ab‐MZF@DMSA. Under the action of ONOO^−^, some thiol groups are oxidized to form disulfide bonds, thereby causing Ab‐MZF@DMSA aggregation.

Aβ induces neuronal oxidative stress, thereby producing ONOO^−^. Next, we tested intracellular Ab‐MZF@DMSA/Gd aggregation. We used the PC12 rat adrenal pheochromocytoma cell line for coculture with Aβ before adding BACE1‐treated Ab‐MZF@DMSA/Gd. Untreated PC12 cells were used as controls. The two groups were examined using TEM. PC12 cells cocultured with Aβ exhibited nanoparticle aggregation, which was not observed in untreated cells (Figure 1E). This finding indicates that Ab‐MZF@DMSA/Gd has ONOO^−^‐sensitive responsiveness and can aggregate in neurons with high ONOO^−^ expression.

As shown in Figure S4 (Supporting Information), the saturation magnetization of BACE1Ab‐MZF@DMSA/Gd+BACE1+ONOO^−^ was 104.7 ± 3.8 emu g^−1^, which was significantly greater than that of untreated Ab‐MZF@DMSA/Gd. This finding shows that the saturation magnetization of the aggregated probes is far greater than that of the dispersed probes.

### 2.3 Ab‐MZF@DMSA/Gd In Vitro Imaging Findings

**Figure 2:**
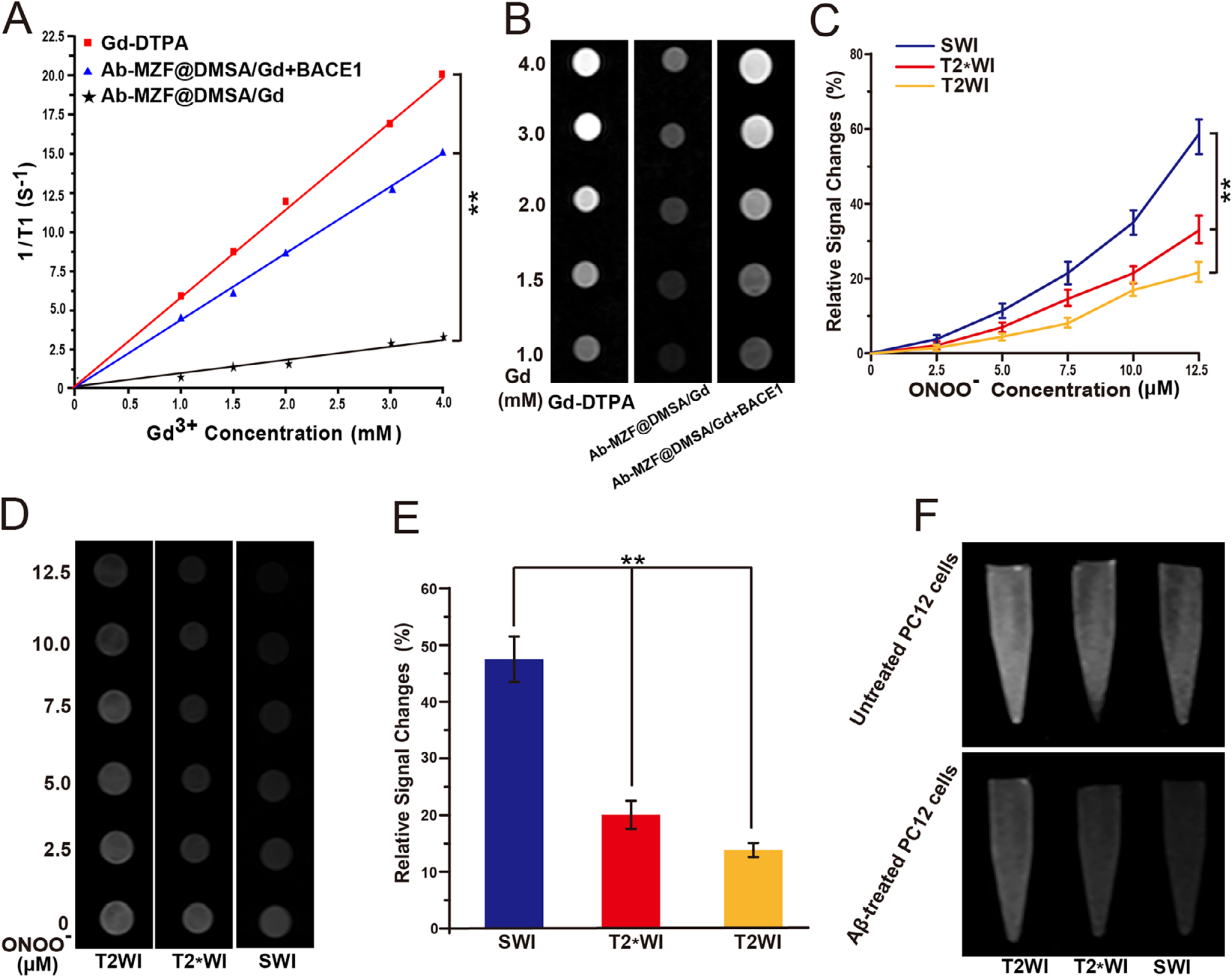
A) T1 relaxation rates (1/T1) and B) T1WI images of Gd‐DTPA, Ab‐MZF@DMSA/Gd and Ab‐MZF@DMSA/Gd+BACE1 at different gadolinium ion concentrations (n = 3). C) Relative SWI, T2_*_WI and T2WI signal changes and D) SWI, T2_*_WI and T2WI images of Ab‐MZF@DMSA/Gd+BACE1 at different ONOO^−^ concentrations (n = 3). E) Quantification of SWI, T2_*_WI and T2WI signal changes and F) representative SWI, T2_*_WI and T2WI images of Ab‐MZF@DMSA/Gd+BACE1 in Aβ‐treated and untreated PC12 cells (n = 3). Statistical significance is indicated (^**^ *p* < 0.01) by Student's *t*‐test.

We measured the T1WI signals of Gd‐DTPA and Ab‐MZF@DMSA/Gd. As shown in **Figure** **2**A,B, Gd‐DTPA is a canonical T1 contrast agent, and its 1/T1 ratio displays a linear relationship with the gadolinium contrast agent. As the gadolinium dose increased, the T1WI signals gradually increased. However, owing to MRET effects, adjacent MZF@DMSA has highly effective quenching effects on the spin magnetic field of Gd‐DTPA, causing its T1WI signals to “switch off”. Therefore, the T1WI signals and r1 values of Ab‐MZF@DMSA/Gd were significantly lower than those of Gd‐DTPA (Figure 2A,B).

After the addition of BACE1, the T1WI signals and r1 of Ab‐MZF@DMSA/Gd gradually increased with an increase in the BACE1 concentration (Figure 2A,B). This is because BACE1 lyses the linker substrate peptide EVNL‐DAEFRHDSGYK in a targeted manner, causing Ab‐MZF@DMSA/Gd lysis. Thus, the distance between the MZF@DMSA and Gd‐DTPA increased significantly. MZF@DMSA no longer interferes with the spin of Gd‐DTPA, allowing the T1WI signal of Gd‐DTPA to be “switched on”. Therefore, the T1WI signals gradually enhanced. Subsequently, we observed almost no significant increase in the T1WI signal intensity of Ab‐MZF@DMSA/Gd after the addition of other proteases (Figure S5, Supporting Information). These results demonstrate the high sensitivity of Ab‐MZF@DMSA/Gd toward BACE1 and that its T1WI signals can be selectively switched on in the presence of BACE1.

Next, we added different concentrations of ONOO^−^ to the BACE1‐treated nanoprobe solution and used SWI to evaluate the ONOO^−^ responsiveness of Ab‐MZF@DMSA/Gd. As shown in Figure 2C,E, the changes in the SWI signal intensity of Ab‐MZF@DMSA/Gd tended to increase linearly with increasing ONOO^−^ concentration. This effect occurs because ONOO^−^ can induce probe aggregation and magnetic exchange coupling increases as the distance between the aggregated probes decreases, thereby resulting in a greater SWI enhancement effect. We also detected changes in the T2_*_WI and T2WI signal intensities after the probe responded to ONOO^−^. We found that the changes in SWI signal intensity were more significant than those in T2_*_WI and T2WI signal intensities (Figure 2C,D). This implies that SWI is more sensitive to magnetic susceptibility changes, causing changes in the SWI signal of the nanoprobes to ONOO^−^.

We next measured the ability of Ab‐MZF@DMSA/Gd to detect intracellular ONOO^−^. MRI was conducted after the BACE1‐treated Ab‐MZF@DMSA/Gd was incubated with Aβ‐treated PC12 cells. We observed that the changes in SWI, T2_*_WI, and T2WI signal intensities of Aβ‐treated PC12 cells were significantly greater than those of untreated PC12 cells and that the changes in the SWI signal intensity were greater than those in the T2_*_WI and T2WI signal intensities (Figure 2E,F). This finding indicates that Ab‐MZF@DMSA/Gd can detect the presence of intracellular ONOO^−^, thereby allowing the sensitive monitoring of intracellular oxidative stress.

### 2.4 In Vitro Uptake of Ab‐MZF@DMSA/Gd by BBB Cells

To evaluate the targeting efficiency, we used brain capillary endothelial cells (BCECSs) that express RAGE on their surfaces as an in vitro BBB model.^[^ ^35^ ^]^

We used a Transwell system to measure the in vitro BBB penetration ability of the nanoprobes (Figure S6, Supporting Information). Fluorescein isothiocyanate (FITC)‐labeled Ab‐MZF@DMSA/Gd and MZF@DMSA/Gd were used to measure the uptake in PC12 cells. The results showed that Ab‐MZF@DMSA/Gd in the upper layer could effectively penetrate BCECs and be taken in by PC12 cells in the lower layer, and that the intracellular fluorescence signal significantly increased. Conversely, the fluorescence of the PC12 cells in the MZF@DMSA/Gd‐group and free antibody pretreatment group was significantly lower (Figure S7, Supporting Information). This finding proves that the Ab peptide modification can increase the BBB penetration capability of the nanoprobe, thereby resulting in cellular uptake.

### 2.5 In Vivo Biological Distribution of Ab‐MZF@DMSA/Gd

According to literature, nanoparticles are mainly absorbed by the reticuloendothelial system.^[^ ^36^, ^37^ ^]^ We observed and measured T2WI signal intensities in the liver and spleen before and after Ab‐MZF@DMSA/Gd injection. Compared to those before injection, the T2WI signal intensities in the liver and spleen after nanoprobe injection gradually decreased and were the lowest after 24 h. These findings indicated that nanoprobe aggregation in the liver and spleen peaked at this time (Figure S8A,B, Supporting Information). This reflects the phagocytosis of nanoprobes by the reticuloendothelial system in the liver and spleen, causing substantial enrichment of the nanoprobes in the liver and spleen and resulting in decreased T2WI signals.

Subsequently, we injected Ab‐MZF@DMSA/Gd into the mice via the tail vein. After 12 h, the mouse tissues were homogenized, and ICP‐OES was used to measure the iron concentrations in the heart, liver, spleen, lungs, kidneys, and brain to observe the distribution of the nanoprobe in various mouse organs.

As shown in Figure S9 (Supporting Information), owing to the reticuloendothelial system in the liver and spleen, Ab‐MZF@DMSA/Gd and MZF@DMSA/Gd were mainly distributed in the liver and spleen in vivo. The iron content in the brain tissues of the Ab‐MZF@DMSA/Gd group was significantly higher than that of the MZF@DMSA/Gd group. This indicates that Ab peptide‐modified probes can penetrate the BBB and aggregate in the brain.

At 96 h after injection of Ab‐MZF@DMSA/Gd, gadolinium and iron levels in the brains of 12‐week‐old AD mice were below 0.004% and 0.5%, respectively (Figure S10, Supporting Information), indicating that the nanoprobes were substantially metabolized in the brains within 96 h with no risk of brain deposition on the nanoprobes.

### 2.6 Biocompatibility of Ab‐MZF@DMSA/Gd

Low toxicity and biocompatibility are important considerations in nanoprobe design. Figure S11 (Supporting Information), shows that the hemolysis percentage of Ab‐MZF@DMSA/Gd with iron concentrations ranging from 2.5 to 50 µg mL^−1^ was lower than the international standard of 5%, indicating that the nanoprobe has good hemocompatibility. Next, we performed H&E staining of the major organs of mice 24 h after nanoprobe injection. We found no significant lesions in the major organs of the mice injected with Ab‐MZF@DMSA/Gd compared to those in the control group, which received a PBS injection (Figure S12, Supporting Information). These findings reveal that the nanoprobe does not cause significant toxicity to the heart, liver, spleen, lungs, or kidneys.

The cytotoxicity of Ab‐MZF@DMSA/Gd was measured using the methyl thiazolyl tetrazolium (MTT) assay. After nanoprobes with iron concentrations ranging from 2.5 to 50 µg mL^−1^ were incubated with human neuroblastoma SH‐SY5Y cells and BCECs for 48 h, the viability of the cells was greater than 90% (Figure S13, Supporting Information), and there was no cytotoxicity. These results demonstrated that the nanoprobe has good biocompatibility and low cytotoxicity.

### 2.7 In Vivo Imaging Using the Nanoprobe

We selected 4‐ and 12‐week‐old female FAD4T transgenic AD mice to assess the in vivo imaging capacity of Ab‐MZF@DMSA/Gd; 12‐week‐old wild‐type (WT) mice were used as controls. The nanoprobes were injected into the different groups via the tail vein and performed T1WI, SWI, T2_*_WI, and T2WI before injection, and 2, 6, 12, 24, and 48 h after injection.

**Figure 3:**
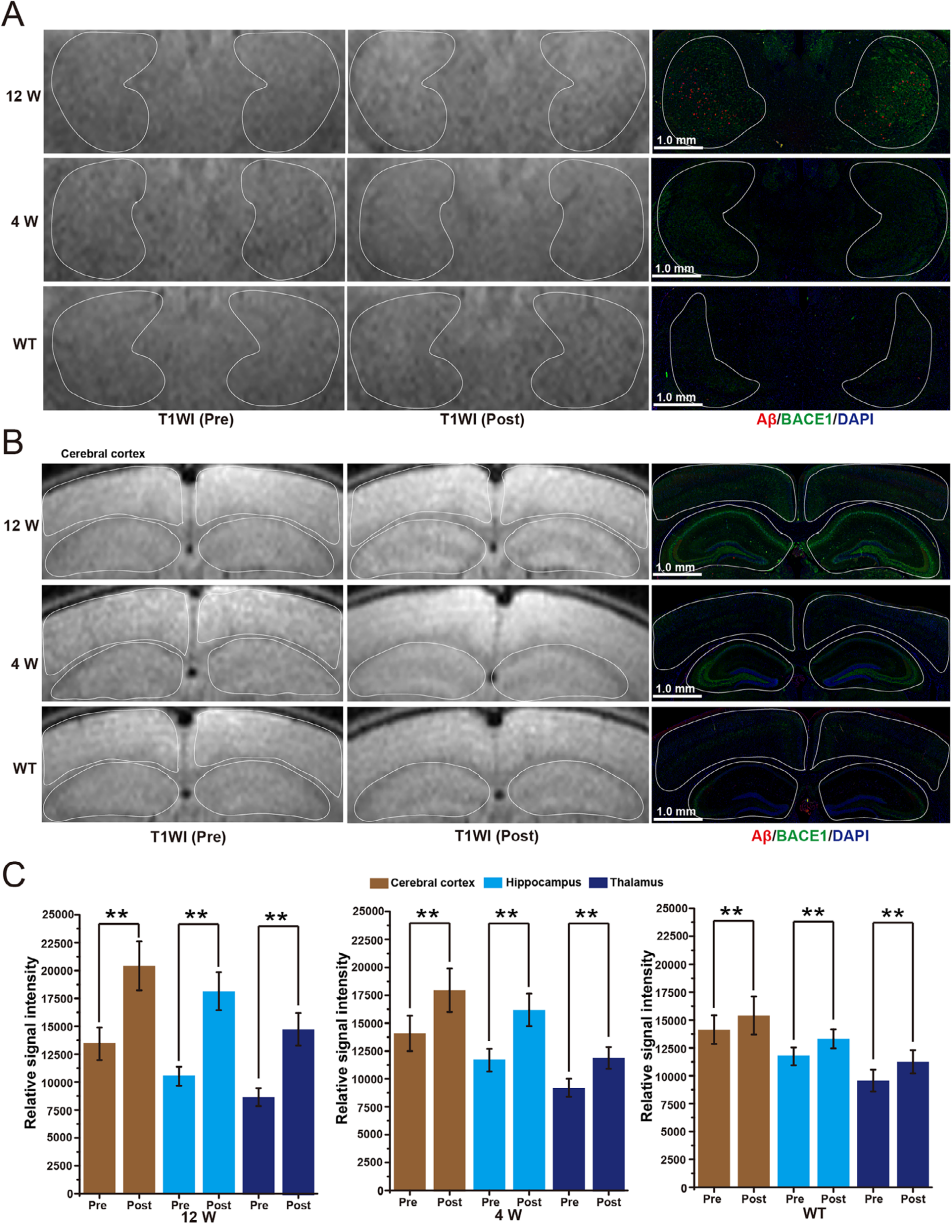
A) Coronal T1WI images and LSCM images of BACE1‐Aβ immunofluorescence of the thalamus in 12‐week‐old AD mice, 4‐week‐old AD mice, and WT mice before and 6 h after Ab‐MZF@DMSA/Gd injection (n = 3). B) Coronal T1WI images and LSCM images of BACE1‐Aβ immunofluorescence of the cerebral cortex and the hippocampus in 12‐week‐old AD mice, 4‐week‐old AD mice, and WT mice before and 6 h after Ab‐MZF@DMSA/Gd injection (n = 3). C) Quantification of T1WI signal intensities of the thalamus, the cerebral cortex, and the hippocampus in 12‐week‐old AD mice, 4‐week‐old AD mice, and WT mice before and 6 h after Ab‐MZF@DMSA/Gd injection (n = 3). Statistical significance is indicated (^**^ *p* < 0.01) by Student's *t*‐test.

Compared to those before the injection of the nanoprobe, the cerebral cortex, thalamus, and hippocampal T1WI signals peaked 6 h after injection in 12‐week‐old AD mice, and the signals gradually decreased thereafter until 48 h after injection (Figure S14, Supporting Information). Changes in the cerebral cortex, thalamus, and hippocampal T1WI signals in the other two groups were also similar. Six hours after nanoprobe injection, the T1WI signal intensity was highest at these three sites in 12‐week‐old AD mice, followed by 4‐week‐old AD and WT mice (**Figure** **3**A–C; Figures S15 and S16, Supporting Information).

In other brain regions among all groups, T1WI signal intensity showed no significant enhancement before and after injection of the nanoprobes (Figures S17 and S18, Supporting Information).

**Figure 4:**
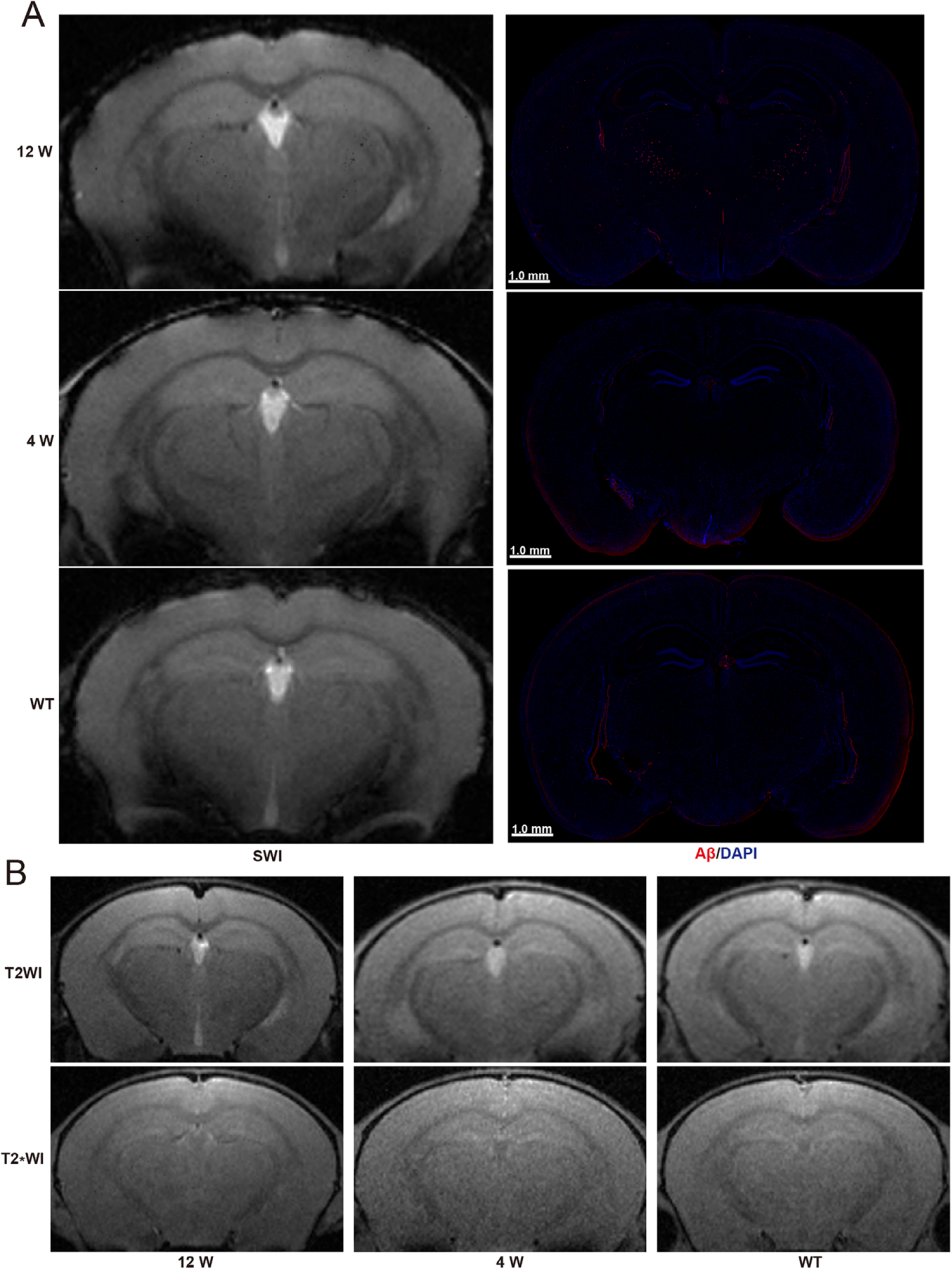
A) Coronal SWI images and LSCM images of Aβ immunofluorescence of the brains in 12‐week‐old AD mice, 4‐week‐old AD mice, and WT mice 12 h after Ab‐MZF@DMSA/Gd injection (n = 3). B) Coronal T2WI and T2_*_WI images of the brains in 12‐week‐old AD mice, 4‐week‐old AD mice, and WT mice 12 h after Ab‐MZF@DMSA/Gd injection (n = 3).

Subsequently, SWI, T2_*_WI, and T2WI were performed. As shown in **Figure** **4**A, there were no significant punctuate low signals in the brains of the different groups before nanoprobe injection. Twelve hours after the nanoprobe injection, punctuate low signals were detected in the cerebral cortex, thalamus, and hippocampus via SWI in 12‐week‐old AD mice (Figure 4A; Figures S19 and S20A, Supporting Information). In contrast, the punctuate low signals of identical sites were blurry and fewer on T2_*_WI and T2WI (Figure 4B; Figure S20A, Supporting Information). No significantly punctuate low signals in these three sequences in 4‐week‐old AD or WT mice (Figure 4; Figure S20A, Supporting Information).

After MR imaging, Aβ/BACE1 immunohistochemistry was performed across the different groups. The results were consistent with the MRI results: BACE1 immunofluorescence in the cerebral cortex, thalamus, and hippocampus increased in the following order: WT mice, 4‐ and 12‐week‐old AD mice (Figure 3; Figure S15, Supporting Information). Correspondingly, BACE1 protein expression levels progressively increased in the brains of WT mice, 4‐ and 12‐week‐old AD mice (Figure S21, Supporting Information).

Red Aβ fluorescent plaques that occurred in 12‐week‐old AD mice were distributed mainly in BACE1 fluorescent regions in the cerebral cortex, thalamus, and hippocampus, and no significant Aβ fluorescent plaques were observed in 4‐week‐old or WT mice (Figures 3 and 4; Figures S15, S21B and S22, Supporting Information).

These results indicate that BACE1 in mouse brains causes targeted lysis of the nanoprobe, resulting in MRET and switching of T1WI signals. As T1WI signal intensity is related to BACE1 levels, T1WI signal intensity was the highest in 12‐week‐old AD mice, which presented the highest BACE1 levels, whereas WT mice, which presented the lowest BACE1 levels, presented the lowest T1WI signal intensity.

We found that BACE1 elevation and Aβ plaques occur mainly in the mouse cerebral cortex, thalamus, and hippocampus. The cortex and hippocampus mainly participate in memory processing, whereas the thalamus is closely associated with memory and learning; these are the main regions where pathological changes in AD occur. Therefore, our nanoprobe can be used for BACE1 imaging detection at the pre‐AD stage before Aβ plaque formation and can detect small Aβ plaques early.

**Figure 5:**
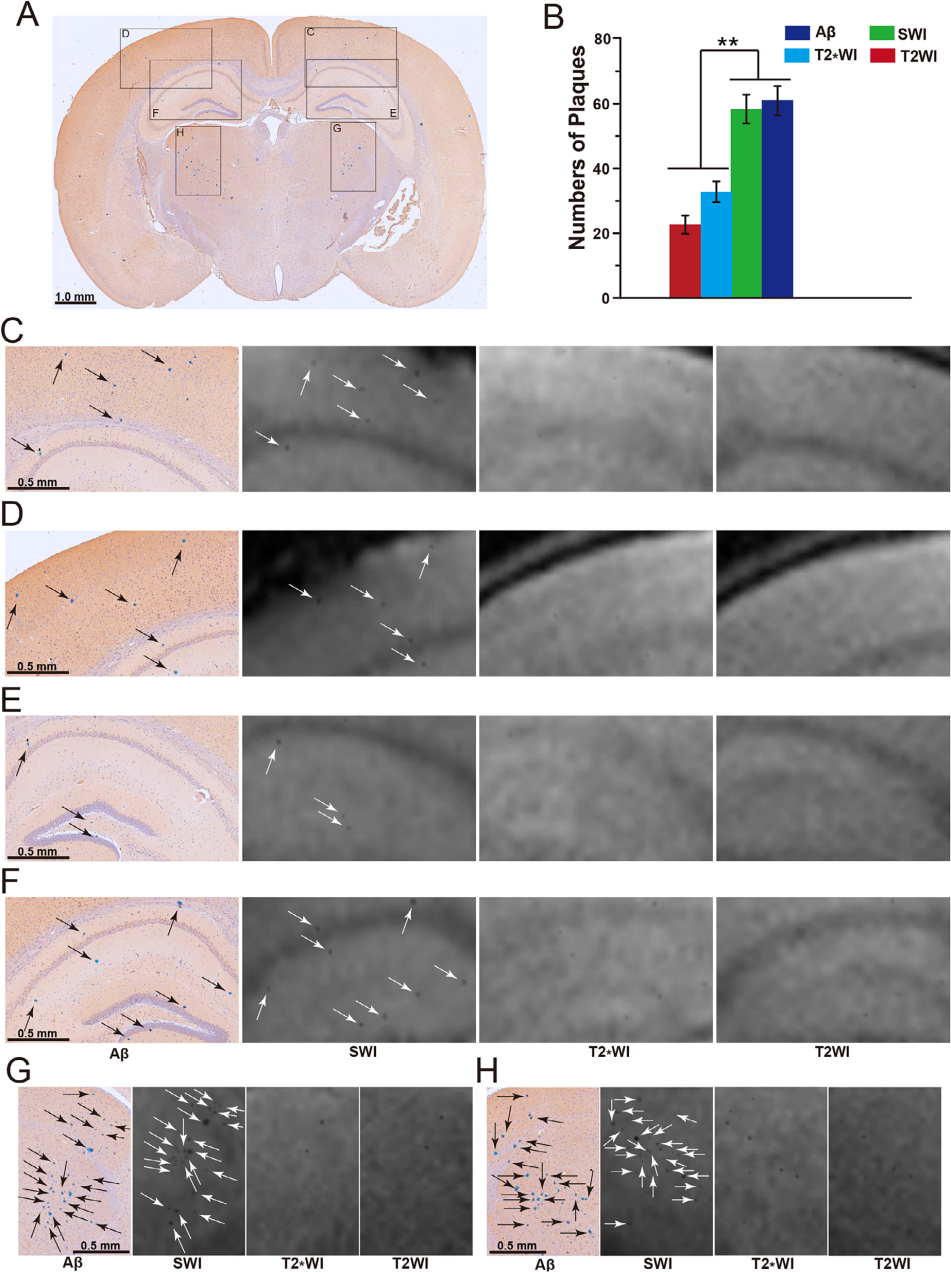
A) Coronal brain sections of 12‐week‐old APP/PS1 mice co‐stained for Aβ‐Prussian blue (n = 3). B) Comparison of plaque counts from Aβ staining, SWI, T2_*_WI, and T2WI imagings in the same coronal brain sections of 12‐week‐old AD mice 12 h after Ab‐MZF@DMSA/Gd injection (n = 3). C–H) Comparison of plaque displays from Aβ and Prussian blue co‐staining (black arrows), SWI (white arrows), T2_*_WI, and T2WI imaging in the same coronal brain sections of 12‐week‐old AD mice 12 h after Ab‐MZF@DMSA/Gd injection. Statistical significance is indicated (^**^ *p* < 0.01) by Student's *t*‐test.

Next, we conducted Aβ‐Prussian blue co‐staining in the same layer of brain tissue that underwent MRI imaging to display Aβ plaques and nanoprobe particles. We found that brown Aβ plaques colocalized with blue nanoprobe particles (Figure S23, Supporting Information). These findings indicate that the nanoprobe can bind to Aβ plaques in a targeted manner. In addition, punctuate low signals in SWI images were generally consistent with Aβ plaques in stained brain tissue sections, and there were no significant differences in the number of plaques (**Figure** **5**). In the T2_*_WI and T2WI images, the punctuate low signals did not completely match, and the number of plaques was significantly lower than that in the Aβ‐Prussian blue co‐staining and SWI images (Figure 5). This finding indicates that the nanoprobe could display small Aβ plaques in SWI imaging.

Western blotting revealed that the expression of inducible nitric oxide synthase (iNOS) in the brain tissues of WT mice, 4‐ and 12‐week‐old AD mice, gradually increased (Figure S24, Supporting Information), and high iNOS expression caused excessive production of ONOO^−^.^[^ ^38^, ^39^ ^]^ Therefore, nanoprobes bound to Aβ plaques cause in situ cross‐linking of thiol groups on surface DMSA through the action of ONOO^−^. This effect causes nanoprobe aggregation, increases magnetic susceptibility, and amplifies SWI signals. This approach allowed the nanoprobe to accurately and sensitively detect small plaques in early AD using SWI.

Finally, we administered nanoprobes to 12‐week‐old AD mice via intrathecal injection (IT). As shown in Figures S25 and S26, Supporting Information, pre‐ and post‐injection T1WI and SWI images revealed no significant differences in imaging efficacy compared to tail vein injection, confirming the diagnostic value of intravenous delivery of Ab‐MZF@DMSA/Gd.

### 2.8 Behavioral Tests in AD Mice

The Morris water maze (MWM) was used to test the learning and memory of different mouse groups. Navigation experiments showed that the time spent swimming on the platform gradually decreased in various groups as the number of training sessions increased. This implies that swim training improved learning and memory in the different groups, and that there was no significant difference in the latency period between the groups (Figure S27, Supporting Information). In the 60‐s spatial exploration experiment, no significant change was noted in the number of platform crossovers across different groups. The results reveal that there was no significant difference in the spatial learning memory of the different groups of mice, and that no spatial learning memory defects occurred in 4‐week or 12‐week‐old AD mice.

In combination with earlier pathological examinations and MR imaging examinations before and after nanoprobe injection, our synthesized nanoprobes could image BACE1 and Aβ plaques in AD regions before the onset of spatial learning memory symptoms in AD mice. Thus, the combination allowed an accurate and sensitive diagnosis of early AD and can be proposed for further treatment.

## 3 Conclusion

Given the role of BACE1, a critical rate‐limiting enzyme for Aβ protein production and Aβ plaques, we developed a smart responsive MR nanoprobe for BACE1 and Aβ plaque imaging. This nanoprobe could penetrate the BBB. T1WI via MRET can be used to detect BACE1 in the brain of AD mice via targeted lysis of the substrate peptide. ONOO^−^ subsequently induces nanoprobe aggregation, which increases magnetic susceptibility and enhances the SWI signals of Aβ plaques. In combination with pathological and behavioral tests, our nanoprobe was shown to have applicability for early AD diagnosis before spatial learning memory defects occur, along with monitoring its pathological progression.

## 4 Experimental Section

### Materials and Syntheses

All materials and synthetic methods are described in the Supporting Information.

### Cells and Animal Models

Human neuroblastoma cells (SH‐SY5Y, RRID:CVCL_0019), Brain capillary endothelial cells (BCECs, RRID:CVCL_A2GE), and rat pheochromocytoma cells (PC12, RRID:CVCL_0481) were purchased from the Shanghai cell bank of the Chinese Academy of Sciences. They were not contaminated. 4‐week‐old and 12‐week‐old FAD^4T^ transgenic mice and 12‐week‐old wild type (WT) mice were purchased from GemPharmatech Co., Ltd. (Nanjing, China). The institutional Animal Care and Use Committees on Animal Care of Tongji Hospital of Tongji University approved the animal protocols (Approval No. (Tong) Ethics Review 2025‐DW‐SB‐293). The mice will be housed in the SPF animal room of the Animal Experiment Center under standard conditions (23 °C ± 2, humidity 50% ± 20, etc.).

BCECs were cultured in DMEM supplemented with 20% FBS, 20 µg mL^−1^ heparin, 2 mmol L‐1 L‐glutamine, 100 µg mL^−1^ epidermal cell growth supplement (Sigma‐Aldrich), 1% of the double‐antibiotic (penicillin and streptomycin) at 37 °C in a 5% CO_2_ ⁄95% air incubator. SH‐SY5Y cells were cultured in Minimum Essential Media/F12 containing 10% FBS, 1% of the double‐antibiotic at 37 °C in a 5% CO_2_ ⁄ 95% air incubator. PC12 cells were cultured in DMEM supplemented with 10% FBS, 1% of the double‐antibiotic at 37 °C in a 5% CO_2_ ⁄95% air incubator. Cells were passaged every 3–4 days. The cells at the logarithmic growth phase were suspended in PBS cells at a density of 1 × 10^7^ cells mL^−1^.

### Characterization of Mn_0.6_Zn_0.4_Fe_2_O_4_, MZF@DMSA/Gd and Ab‐MZF@DMSA/Gd

1. Transmission electron microscopy was used to observe the morphology and size of various samples.
2. Dynamic light scattering was used to measure the size and distribution of various samples.
3. The structural formations of Mn_0.6_Zn_0.4_Fe_2_O_4_ were characterized by X‐ray diffraction.
4. A vibrating sample magnetometer was used to measure the hysteresis curve of various samples at room temperature (300K).
5. The infrared (IR) spectra of various samples were characterized by Fourier transform infrared spectroscopy.
6. The Raman spectra of various samples were characterized by Raman spectroscopy.

### BACE1‐Triggered Gadolinium Ions Release

0.5 mL of MZF@DMSA/Gd was mixed with 0.5 mL of BACE1 at various concentrations (5, 10, 25, 50 µM) (caspase‐3, γ‐GGT, GSH, MMP‐2, AChE, and BChE) solution (10 µM).

After 2 h, all of the samples were placed on a magnet, and the supernatant was collected after magnetic separation. The gadolinium ion concentrations in each supernatant were measured by ICP‐OES.

### ONOO^−^‐Triggered Nanoprobe Self‐Aggregation

1. TEM was used to observe the morphology and size of Ab‐MZF@DMSA/Gd+BACE1 treated with ONOO^−^ at various concentrations (15 µM, 30 µM) or H_2_O_2_ (500 µM), O^2.−^ (100 µM), HCIO (100 µM), OH (100 µM) or Fe^3+^ (200 µM), Mn^2+^ (200 µM), Na^+^ (1000 µM).
2. A vibrating sample magnetometer was used to measure the hysteresis curve of Ab‐MZF@DMSA/Gd+BACE1 treated with ONOO^−^ at room temperature (300K).
3. DLS was used to measure the size and distribution of Ab‐MZF@DMSA/Gd+BACE1 treated with ONOO^−^.
4. PC12 cells were pretreated with 20 µM Aβ aggregates for 12 h. Ab‐MZF@DMSA/Gd (Fe concentration: 20 µg mL^−1^) + BACE1 was incubated with untreated PC12 cells or Aβ‐treated PC12 cells in serum‐free medium for 4 h and then washed three times with PBS. Cells were separated and collected from the culture dish using trypsinization. Then, the samples were centrifuged at 800 rpm for 15 min, washed three times with PBS, pre‐fixed with 2.5% glutaraldehyde at room temperature, observed, and photographed under the TEM.

### MRI Examination of Nanoprobe

1. BACE1‐Induced T1WI Signal Changes. The gadolinium content was determined in Ab‐MZF@DMSA/Gd on an emission spectrometer using ICP‐OES. To assess the performance of the enhanced MR T1WI of Ab‐MZF@DMSA/Gd in different states, two Ab‐MZF@DMSA/Gd samples and one Gd‐DTPA sample with different concentration gradients and in different states were prepared for the measurement of R1, including (1) Ab‐MZF@DMSA/Gd; (2) Ab‐MZF@DMSA/Gd after BACE1 incubation treatment.

2 mL of each solution was placed in the NMR tube. Under 25 °C constant temperature, the T1 value of the contrast agent at different concentration gradients was measured with a Bruker 7.0 T MRI scanner. A plot was made with 1/T1 on the *y*‐axis and Gd^3+^ concentration on the *x*‐axis. The relaxation rate r1 was obtained by linear fitting. Ab‐MZF@DMSA/Gd was mixed with caspase‐3, GGT, GSH, and MMP‐2, respectively. Then, their changes of T1WI signal intensities were measured.

1. ONOO^−^‐Induced SWI, T2_*_WI, and T2WI Signal Changes. To assess the response of Ab‐MZF@DMSA/Gd to ONOO^−^via SWI, T2_*_WI, and T2WI, Ab‐MZF@DMSA/Gd (Gd concentration: 2.5 mM) + BACE1 at different ONOO^−^ concentrations were prepared.

Ab‐MZF@DMSA/Gd (Gd concentration: 2.5 mM) is prepared with BACE1 in Aβ‐treated and untreated PC12 cells in serum‐free medium for 4 h. Washed three times with PBS, and cells were separated and collected from the culture dish via trypsinization.

2 mL of each solution was placed in the NMR tube. Under 25 °C constant temperature, a Bruker 7.0 T MRI scanner was used for SWI, T2_*_WI, and T2WI sequence scanning of different samples.

### Assement of In Vitro BBB Penetration of Ab‐MZF@DMSA/Gd

In vitro BBB model was constructed by the BCECs monolayer transwell system to study the BBB crossing ability of nanoprobes. Briefly, BCECs cells were cultured, seeded onto Transwell plates with permeable support to mimic the BBB in vitro. After grown for 11 days, the cell monolayer permeability was assessed by a 4 h water‐leaking test, and TEER was higher than 240 Ω cm^2,^ indicating the validity of the in vitro BBB model. The culture inserts were transferred to a 6‐well plate that was previously seeded with PC12 cells.

FITC‐labeled nanoprobe was prepared. 1 mL FBS‐free medium containing different nanoprobes was added to the apical compartment, and 2.5 mL medium to the basal compartment. The BBB permeability of each sample was compared with the fluorescence intensity of PC12 cells by fluorescence microscopy. Flow cytometry was also performed to quantify the fluorescence intensity and the percentages of fluorescence‐positive cells of the PC12 cells.

### In Vivo MRI Scan

 

1. T2WI Scan of Ab‐MZF@DMSA/Gd Distribution in the Liver and Spleen. 3 WT mice weighing ≈20 g were selected. An Ab‐MZF@DMSA/Gd probe solution (0.3 mL at 1 mg mL^−1^, 2.25 mg Fe kg^−1^, 0.038 mmol Gd kg^−1^) was injected via tail vein. Each mouse underwent T2WI scans on a 7.0T MRI before injection and at 2, 6, 12, 24, and 48 h post‐injection. to monitor liver and spleen signal intensity changes. During the scan, the mice were initially anesthetized with 2.5% isofurane in 75% NO_2_ blended with 22% O_2,_ which was later reduced to 1% isofurane for maintaining anesthesia.

MRI scan sequence parameters: T2WI spin‐echo: Repetition time (TR) = 3000.0 ms, TE = 30.0 ms, Repetitions = 1, field of view (FOV) = 45.0 mm × 35.0 mm; slice thickness = 1.0 mm/1.0 mm, Matrix = 256 × 256, Scan time = 3 min and 12 s.

1. T1WI, SWI, T2_*_WI, T2WI Scan of Brain. An Ab‐MZF@DMSA/Gd probe solution (0.3 mL at 1 mg mL^−1^, 2.25 mg Fe kg^−1^, 0.038 mmol Gd kg^−1^) was injected via tail vein. Each mouse underwent brain T1WI, SWI, T2_*_WI, and T2WI scans on a 7.0T MRI before injection and at 2, 6, 12, 24, and 48 h postinjection to monitor the intensity changes. During the scan, the mice were initially anesthetized with 2.5% isofurane in 75% NO_2_ blended with 22% O_2,_ which was later reduced to 1% isofurane for maintaining anesthesia.

Mice subjected to subarachnoid injection:After anesthetizing the mice, fix them in a prone position with 30° neck flexion to expose the occipital bone‐C1 space. Insert the needle vertically to a depth of 3–3.5 mm until cerebrospinal fluid reflux is observed, then slowly inject 5 µL of nanoprobe solution. Keep the needle in place for 1 min before withdrawal. After the procedure, apply pressure to stop bleeding and administer antibiotic ointment. Each mouse underwent brain T1WI, SWI, scans on a 7.0T MRI before injection and at 6 h postinjection to monitor the intensity changes.

The acquisition sequences and parameters are as follows:

T1WI: TR = 1500.0 ms, TE = 6.5 ms, Repetitions = 1, FOV = 45.0 mm × 35.0 mm, slice thickness = 1.0 mm/1.0 mm, Matrix = 256 × 256, Scan time = 2 min and 24 s.

SWI: TR = 600.0 ms; TE = 18.0 ms; Repetitions = 1, FA = 30.0°; FOV = 20.0 mm × 20.0 mm; Matrix = 256 × 256; Scan time = 10 min 52 s and 400 ms.

T2WI: TR = 3000.0 ms, TE = 30.0 ms, Repetitions = 1, FOV = 45.0 mm × 35.0 mm, slice thickness = 1.0 mm, Matrix = 256 × 256; Scan time = 3 min and 12 s.

T2_*_WI: TR = 500.0 ms; TE = 10.0 ms; Repetitions = 1, FA = 30.0°; FOV = 45.0 mm × 35.0 mm; Matrix = 256 × 256; Scan time = 2 min and 52 s.

The MRI images were analyzed by Image J software (National Institutes of Health, Bethesda, USA) to calculate the numbers by Aβ amyloid plaques, which were labeled by nanoprobes and manifested as dark intensity in SWI, T2_*_WI, and T2WI in 3 serial sections of each brain, and the T1WI magnetic resonance signal of the tumor sites was measured.

### Ab‐MZF@DMSA/Gd Distribution in the Mice

The concentrations of iron in the heart, liver, spleen, lungs, kidneys, and brains of the mice at 12 h after the injection of Ab‐MZF@DMSA/Gd or MZF@DMSA/Gd through the tail vein were determined by ICP‐OES.

The concentrations of gadolinium and iron in the brains of the mice at 96 h after the injection of Ab‐MZF@DMSA/Gd through the tail vein were determined by ICP‐OES.

### In Vitro Hematological Analysis

Blood was obtained from healthy New Zealand rabbits and anticoagulated with potassium oxalate. Ab‐MZF@DMSA/Gd detected were divided into five concentration groups. 0.9% saline and distilled water were used as negative and positive controls, respectively. Then, 0.2 mL of diluted anticoagulated blood was added to each tube preheated. After incubation for 60 min, the tubes were centrifugated for 5 min. Next, the supernatant fluid was assembled, and OD values were measured at 545 nm by UV–vis spectrophotometry. The hemolysis rate (HR) was calculated as follows: HR(%) = (OD of the experimental group‐OD of negative control group)/(OD of the positive control group‐OD of the negative control group) × 100%.

### In Vitro Cytotoxicity

SH‐SY5Y and BCECs cells were seeded in 96‐well cell culture plates, with each well consisting of 1×l0^4^ cells, and then cultured in an incubator supplied with 5% CO_2_ at 37 °C for 24 h. Then, the cells were treated with different Ab‐MZF@DMSA/Gd concentrations in the media for 24 h. The cell viability of different treatments was calculated by the standard MTT assay.

### Histopathological Analysis

 

1. H&E Staining of Main Organs in the Mice. 24 h after the injection of Ab‐MZF@DMSA/Gd or PBS through the tail vein, the mice in each group were sacrificed, and the organs were separated from the bodies to measure the H&E staining. The heart, liver, spleen, lung, kidney, and tumors were fixed in formalin and processed in paraffin. Then, the tissues were sliced at 4 µm thickness for H&E staining.
2. Immunofluorescence Examination of the Brains in the Mice. Mice were perfused with PBS, and the brains were fixed with 4% paraformaldehyde (PFA) for 2 days, followed by incubation in 30% sucrose solution for 2 days until the brains sunk to the bottom. Sucrose‐downed mouse brains were sagittally sectioned by a sliding microtome at 40 mm thick, unless otherwise indicated. For the immunostaining, at least ten brain sections encompassing a whole hemisphere of each mouse were incubated with 0.3% Triton X‐100, 0.3% Donkey serum, and 3% H_2_O_2_ for 20 min at room temperature, to increase antibody permeability and to remove endogenous peroxidase activity. Sections were washed with TBS and then incubated with 0.3% Triton X‐100, 5% Donkey serum, and primary antibody for overnight at 4 °C. Primary antibodies used for immunostaining are Aβ and BACE1. For light microscopic analysis, the bound primary antibodies were detected by biotin‐conjugated secondary antibodies, followed by incubation with avidin‐biotin‐peroxidase complex and by subsequent development of the bound peroxidase activity with 3,30 ‐diaminobenzidine. Stained sections were dehydrated with increasing concentration of ethanol and mounted with Cytoseal. Staining was visualized with a microscope, and photomicrographs were taken with an attached digital camera and associated software. For confocal microscopic analysis, the primary antibody‐bound sections were incubated with AlexaFluor 488 or 568‐conjugated secondary antibody. DAPI (4′,6‐diamidino‐2‐phenylindole) was used for nuclear counter staining, and DABCO (1,4‐diazabicyclo[2.2.2]octane) was used for mounting. Fluorescence images were captured by confocal microscopes.

Whole brains (5 brain sections/mouse) were analyzed for Aβ plaque numbers and Ab plaque‐covered areas by utilizing Analyze Particles method of ImageJ.

### Aβ‐Prussian Blue Double Staining for the Brains in the Mice

The brains were removed from the agarose gel and soaked in 30% sucrose at 4 °C until the tissue sank. Brain tissues were sectioned at 40 µm thickness and were mounted on the glass slides. All sections were first stained overnight with a combination of monoclonal anti‐Aβ antibodies at a dilution of 1:50. Then, the immunolabeled sections were treated with biotin‐labeled anti‐mouse IgG secondary antibodies at a 1:50 dilution for 1 h. Sections were further treated with a working solution of DAB chromogenic substrate solution for 30 min in the dark to ensure better color development. Sections were rinsed with water, and a mixture of 10% potassium ferrocyanide and 20% hydrochloric acid solution was applied to each section for 1 h. Sections were rinsed with water and coverslipped with mounting medium. The sections were examined under bright or fluorescence view of a high‐power microscope in order to locate the iron oxide, Aβ plaques.

### Western Blot Analysis

4‐week‐old, 12‐week‐old AD mice and WT mice were sacrificed, and brain tissues were respectively lysed in RIPA lysis buffer on ice. PMSF was added as the protease inhibitor (1:100, v/v). Samples were added to 5× Loading buffer and heated at 90 °C for 10 min. An equal amount of proteins (30 g) underwent 10% SDS‐PAGE and then transferred to polyvinylidene difluoride (PVDF) membrane. The membrane was blocked in TBST containing 5% non‐fat milk for 1 h. Membrane was incubated with primary antibody (iNOS, Proteintech; BACE1, Abcam) diluted in TBST at 4 °C overnight. After 3 washings in TBST, the PVDF membrane was incubated with appropriate horseradish peroxidase‐conjugated secondary antibodies (1:7500) for 1 h at room temperature. The immunoreactive bands were developed with the ECL western blotting system. β‐actin was used as a loading control. Parameters for loading buffer electrophoresis were set as: 150 V, 30 min. Parameters of electrophoresis for transferring the membrane are 200 mA, 70 min.

### Morris Water Maze Test

The place navigation test was conducted to assess mice's learning and memory of the water maze over 4 days, with two training sessions each morning and afternoon. Mice were randomly released from the center of a quadrant into the pool. If a mouse climbed onto the platform and stayed for over 5 s, it was removed and dried. The software recorded swimming time (escape latency) and trajectory. Mice not finding the platform within 60 s were guided there, with latency recorded as 60 s. All operations were done by one person to reduce odor interference, with a 15‐min interval between sessions. The escape latency and strategy for nine training trials were recorded.

The spatial exploration test on day 5 assessed mice's memory of the platform's location. The platform was removed, and mice were released from the second quadrant's center (facing the pool wall) in the afternoon. The time spent in the original platform quadrant, crossings over the platform, and swimming trajectory within 180 s were recorded.

### Statistical Analysis

Statistical analyses were performed using SPSS 23.0 software (IBM, USA). Results were expressed as mean ± standard deviation. The significance of the difference was analyzed by Student's t‐test and one‐way analysis of variance (ANOVA). Significant differences were indicated by ^*^ *p* < 0.05; ^**^ *p* < 0.01.

## Conflict of Interest

The authors declare no conflict of interest.

## Supporting information



Supporting Information

## Data Availability

The data that support the findings of this study are available from the corresponding author upon reasonable request.
