# Supplementary material for: T1WI‐SWI Dual Modal Magnetic Resonance Nanoprobes for Accurate Diagnosis of Early Stage Alzheimer's Disease
Source: Adv Sci (Weinh). 2025 Oct 27;13(1):e10298. doi: 10.1002/advs.202510298 (PMC12767124; doi:10.1002/advs.202510298)
Supplement: Supplementary file 1 — Supporting Information [file ADVS-13-e10298-s001.docx]

**T1WI-SWI Dual Modal Magnetic Resonance Nanoprobes for Accurate Diagnosis of Early Stage Alzheimer’s Disease**

*Minghua Li, Aijun Shen, Xiaolong Gao, Chao Lin, Zongliang Huang, Qi Lv, Junjun Tang, Xiaolong Ma, Jiong Ni, Ju Tian, Jiaqi Wu, Xiaowen Xu, Wei Wang and Peijun Wang**

Dr. M. Li, Dr. A. Shen, Dr. Z. Huang, Dr. Q. Lv, Dr. J. Tang, Dr. X. Ma, Dr. J. Ni, Dr. J. Tian, Dr. J. Wu, Dr. X. Xu, Dr. W. Wang, Prof. P. Wang

Department of Medical Imaging, Tongji Hospital, School of Medicine, Tongji University, Shanghai, 200092, P.R. China

Institute of Medical Imaging Artificial Intelligence, Tongji University School of Medicine, Shanghai, 200092, P.R. China

1. mail: [wangpeijuntjyy@sina.com](mailto:wangpeijuntjyy@sina.com)

Dr. X. Gao

Department of Radiology, Luodian Hospital, Shanghai University, Shanghai 201908, P.R. China

Department of Radiology, Baoshan District, Luodian Hospital, Shanghai 201908, P.R. China.

Prof. C. Lin

Department of Periodontology, School and Hospital of Stomatology, Shanghai Engineering Research Center of Tooth Restoration and Regeneration, Tongji University, Shanghai 200072, PR China

School of Medicine, Tongji University, Shanghai 200092, PR China

**1. Experimental Section**

**Materials and Instruments**

dimercaptosuccinic acid (DMSA), peroxynitrite (ONOO^-^), epidermal cell growth supplement (Sigma-Aldrich, USA), EVNL-DAEFRHDSGYK (Zheng TAI Biochemical, China), Ab peptide (KLVFFAED) (Sangon Biotech, China), oleic acid, oleylamine, 1,2-Hexadecanediol (Aladding Shanghai, China), recombinant mouse BACE1 protein, recombinant anti-beta amyloid 1-42 antibody, recombinant anti-BACE1 antibody (Abcam, UK), methyl thiazolyl tetrazolium (MTT) kit, Prussian blue staining kit (Nanjing KeyGen Biotech, China), dulbecco’s modifified Eagle’s medium (DMEM), RPMI 1640, fetal bovine serum (FBS), phosphate Buffered Saline (PBS), and trypsin (Gibico, USA), Transwell system (Corning, USA),

Raman spectrometer (invia-reflex, Renishaw, UK), XRD diffractometer (Rigaku D/Max-2550VB3+, Japan), Avance 500 MHz NMR spectrometer (Bruker BioSpin, Switzerland), UV-vis spectroscopy (UV-Vis-NIR, Cary 5000, Agilent, USA), transmission electron microscopy (TEM) (JEM-2010F, JEOL, Japan), flow cytometry (C6, BD, USA), fluorescence microscope (Ti-S, Nikon, Japan), laser scanning confocal microscopy (LSCM) (TCS SP5, Leica, Germany), 7.0 T small animal MRI (BioSpec 70/20, Bruker, Germany), dynamic light scattering (Malvern Autosizer 4700, U.K.), plasma optical emission spectrometer (ICP-OES) (SPECTROARCOS, Spectro, Germany), transendothelial electrical resistance (TEER) (ERS-2, Millipore, America), Morris water maze and software analysis system (Shanghai Mobile Datum, China), a high power microscope (Axiophot 2, Carl Zeiss AG, Germany).

**Synthesis and Preparation of Ab-MZF@DMSA/Gd**

*Synthesis of Mn_0.6_Zn_0.4_Fe_2_O_4_*

We used liquid phase thermal decomposition to prepare the Mn0.6Zn0.4Fe2O4.^[1-3]^

*Preparation of MZF@DMSA Nanoparticle*

Mn0.6Zn0.4Fe2O4 in 1 mL of toluene and 12.5 mg of DMSA in 1 mL of MeOH were mixed and shaken for 12 hours. Next, the mixture was centrifuged at 3000 r.p.m. for 3 minutes and the precipitates were collected and dried under the vacuum. Then, 1 mL of deionized water was added and the pH for the solution was adjusted to a pH of 8. The solution was purified via a ‘PD MiniTrapTM G-10’ column.

*Synthesis of MZF@DMSA/Gd*

The BACE1 substrate peptide (10 μM) was crosslinked with the Gd-DTPA copolymer via 1-ethyl-3-(3-dimethylaminopropyl)carbodiimide (EDC)/N-hydroxysuccinimide (NHS) to form a peptide-Gd complex. The complex was then mixed with MZF@DMSA at a 1:1 molar ratio and reacted at room temperature for 6 hours to form the MZF@DMSA/Gd probe.

*Synthesis of Ab-MZF@DMSA/Gd*

The klvffaed peptide (5 μM) was conjugated to the surface of the probe via a thiol-maleimide reaction under stirring at room temperature for 4 hours. The resulting product was then purified by magnetic separation and dispersed in saline for further use.

**References**

1. K. Cheng, S. Peng, C. Xu, S. Sun, J. Am, “Porous Hollow Fe_3_O_4_ Nanoparticles for Targeted Delivery and Controlled Release of Cisplatin,” *J. Am. Chem. Soc.* **2009**, 131, 10637.

[2] J. Lu, S. Ma, J. Sun, et al., “Manganese ferrite nanoparticle micellar nanocomposites as MRI contrast agent for liver imaging,” *Biomaterials.* **2009**, 30, 2919.

[3] S. Sun, H. Zeng, D.B. Robinson, et al., “Monodisperse MFe_2_O_4_ (M = Fe, Co, Mn) Nanoparticles,” *J. Am. Chem. Soc.* **2004**, 126, 273.

1. **Figures**


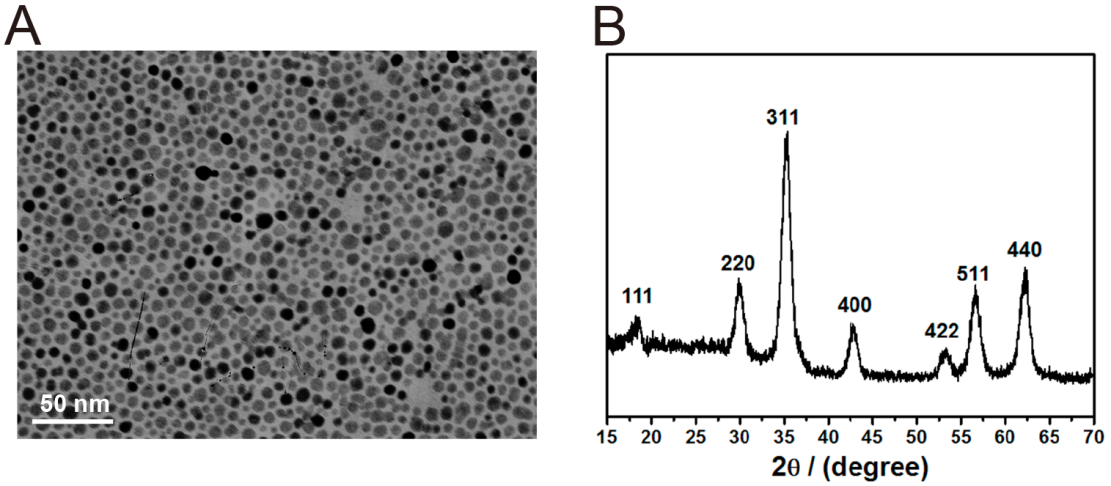


**Figure S1.** A) TEM image of Mn_0.6_Zn_0.4_Fe_2_O_4_ particles. B) XRD image of Mn_0.6_Zn_0.4_Fe_2_O_4_.


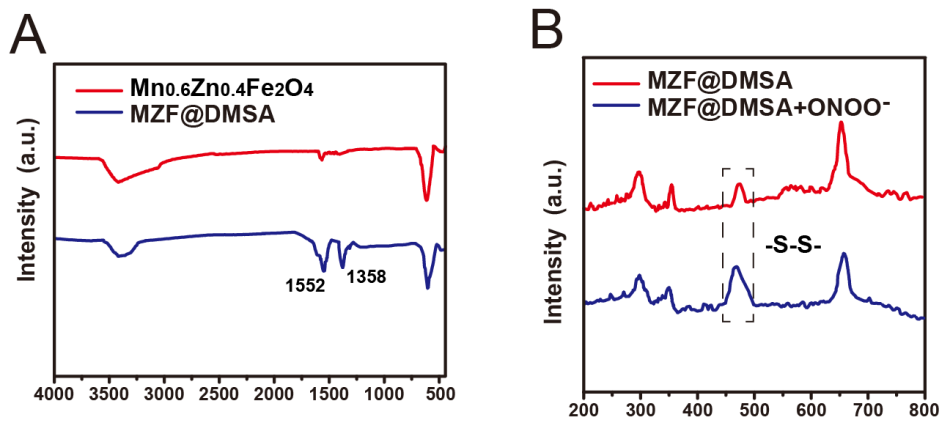


**Figure S2.** A) Infrared spectrum of Mn_0.6_Zn_0.4_Fe_2_O_4_ and MZF@DMSA. B) Raman spectrum of MZF@DMSA and MZF@DMSA+ONOO^-^.


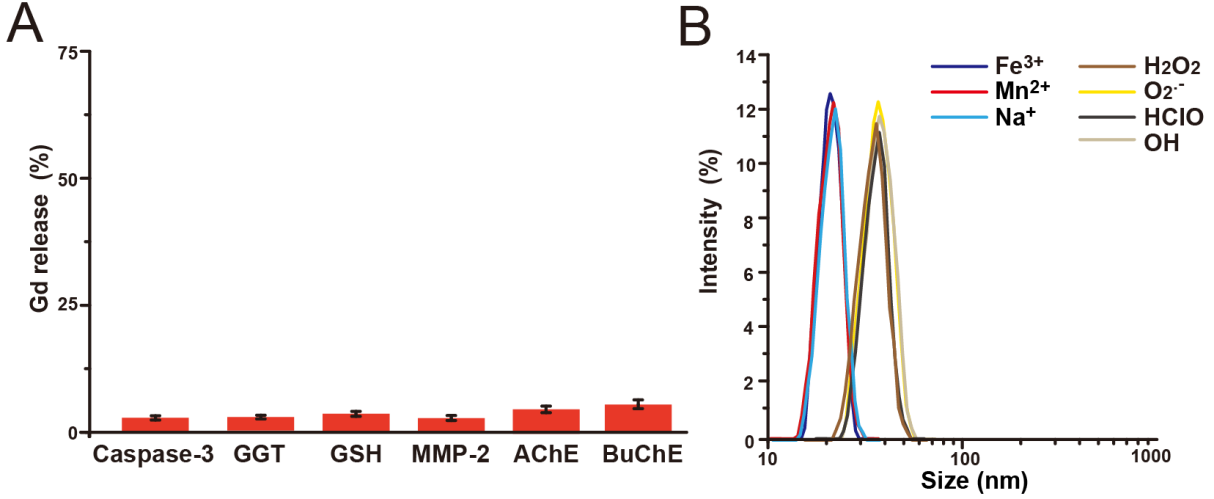


**Figure S3.** A) Gadolinium ions release ratios after adding different enzymes with a concentration of 10 μM (n = 3). B) DLS measurements of Ab-MZF@DMSA/Gd+BACE1 treated with different metal ions and ROS (n = 3). Statistical significance is indicated by Student’s t-test.


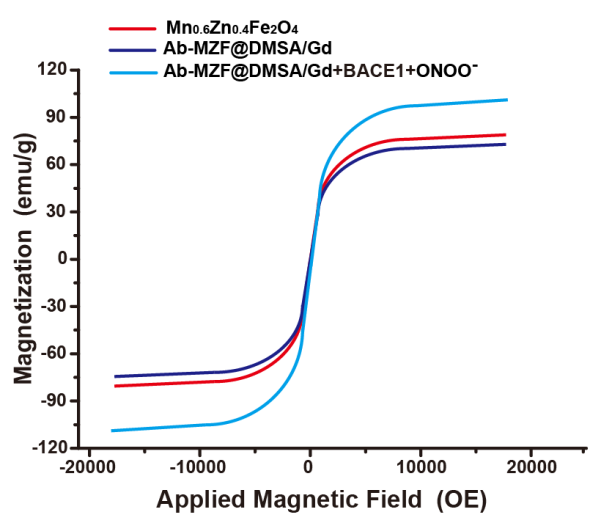


**Figure S4.** Magnetic curves of Mn_0.6_Zn_0.4_Fe_2_O_4_, Ab-MZF@DMSA/Gd and Ab-MZF@DMSA/Gd+BACE1+ONOO^-^ (n = 3).


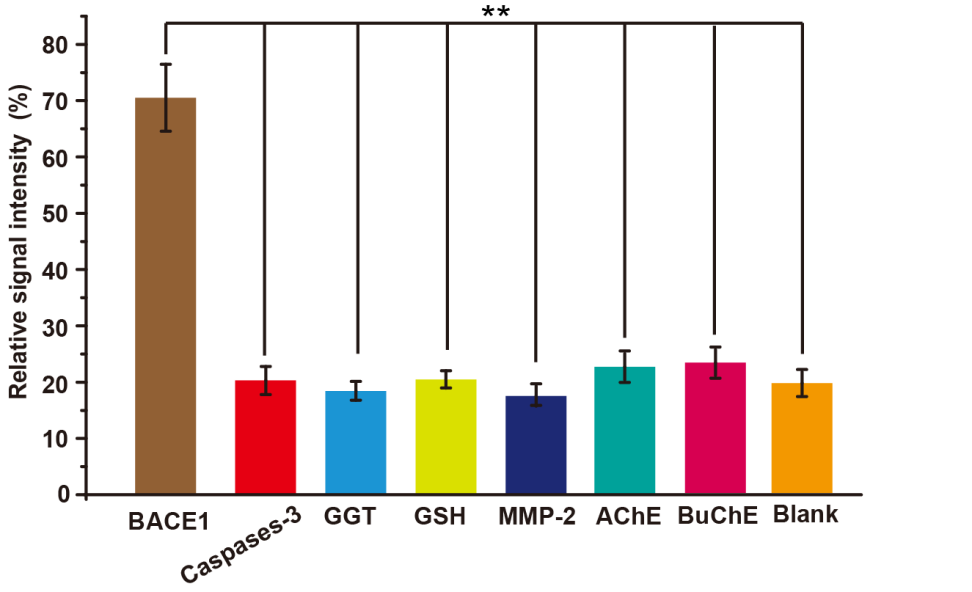


**Figure S5.** Quantification of T1WI signal intensities of Ab-MZF@DMSA/Gd after adding different enzymes with a concentration of 10 μM (n = 3). Statistical significance is indicated ( **p < 0.01) by Student’s t-test.


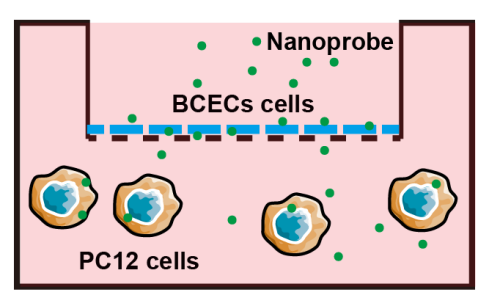


**Figure S6.** Illustration of the BCECs monolayer Transwell system to simulate the BBB *in vitro*.


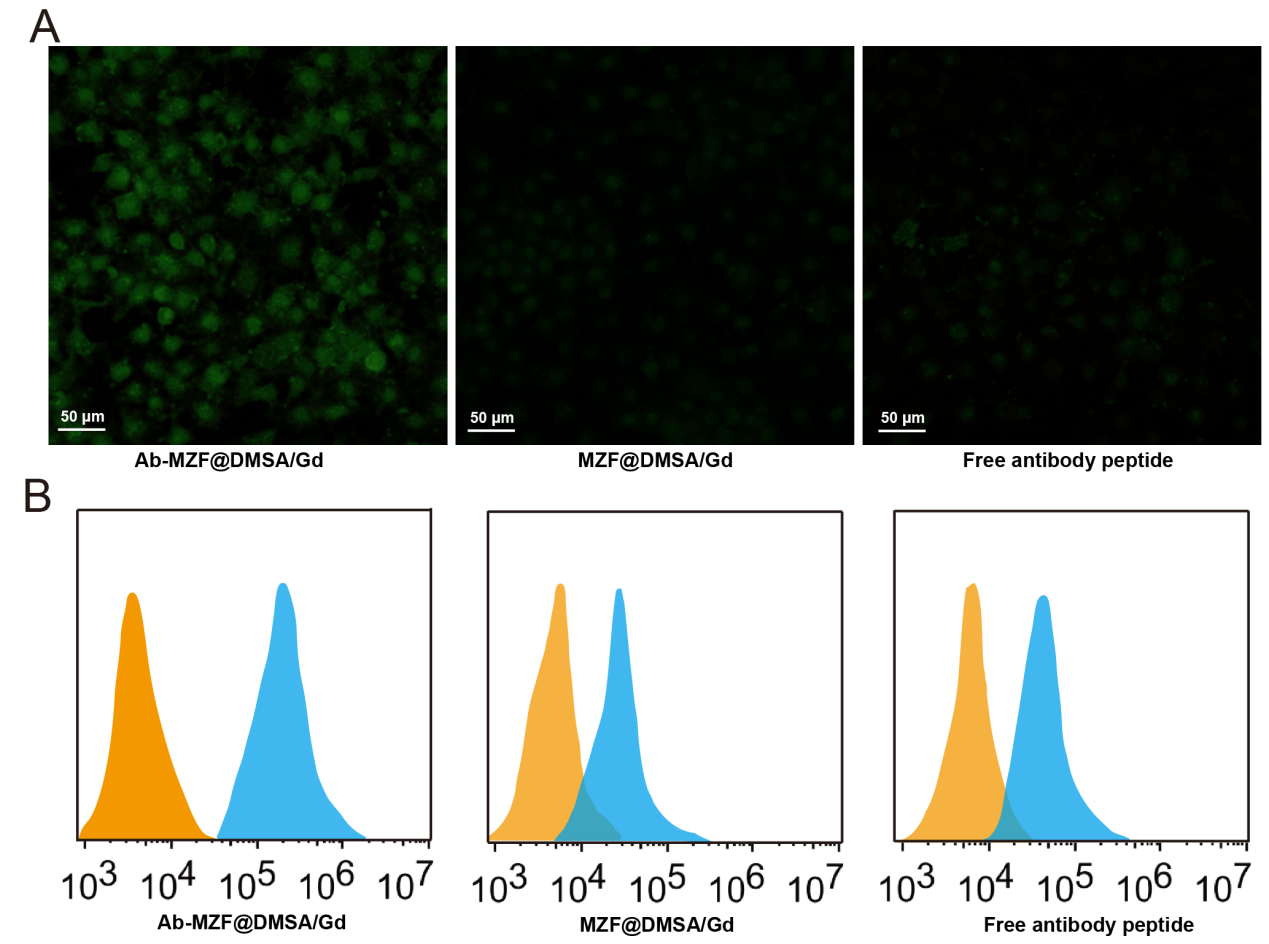


**Figure S7.** A) Confocal images and B) flow cytometry measurements of *in vitro* BBB penetration of nanoprobes by PC12 cells via Transwell system.

**
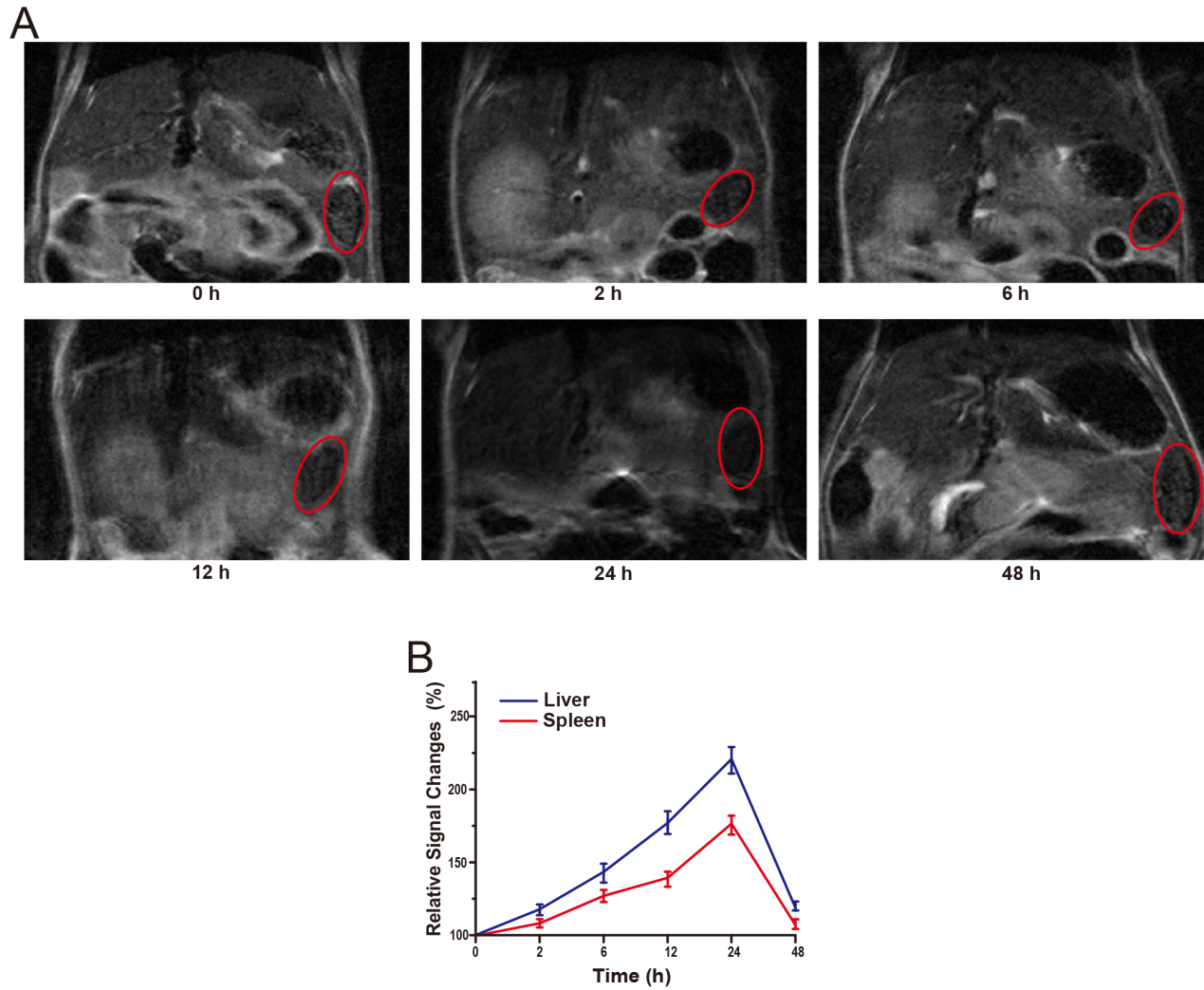
Figure S8.** A) T2WI images B) relative signal changes of the livers and spleens (red circles) at various time points before and after Ab-MZF@DMSA/Gd nanoparticles injection (n = 3).


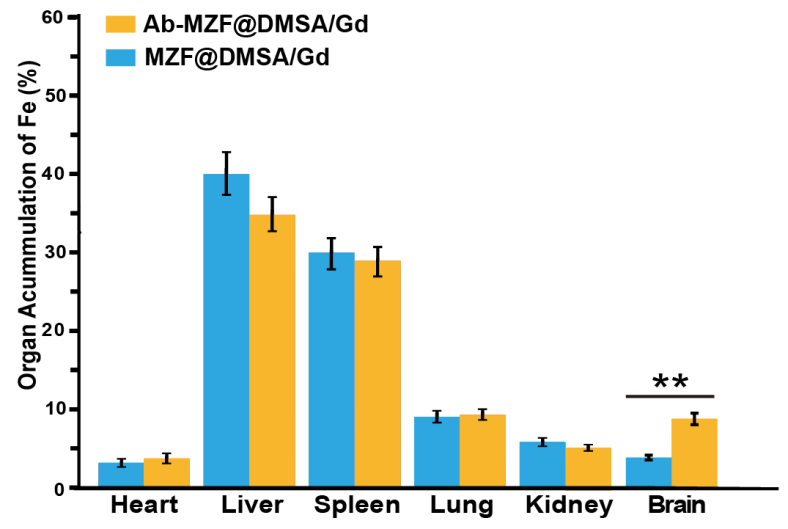


**Figure S9.** Main organs distribution of Iron 12 hours after intravenous injection of Ab-MZF@DMSA/Gd. (n = 3). Statistical significance is indicated ( **p < 0.01) by Student’s t-test.


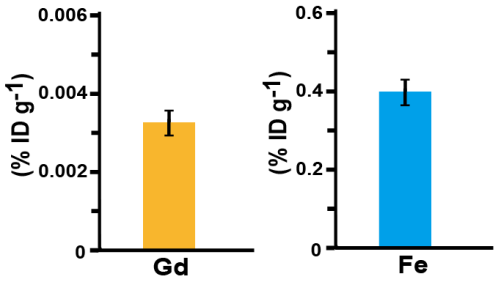


**Figure S10.** Gadolinium and Iron levels in the brains of 12-week-old AD mice 96 hours after intravenous injection of Ab-MZF@DMSA/Gd (n = 3).


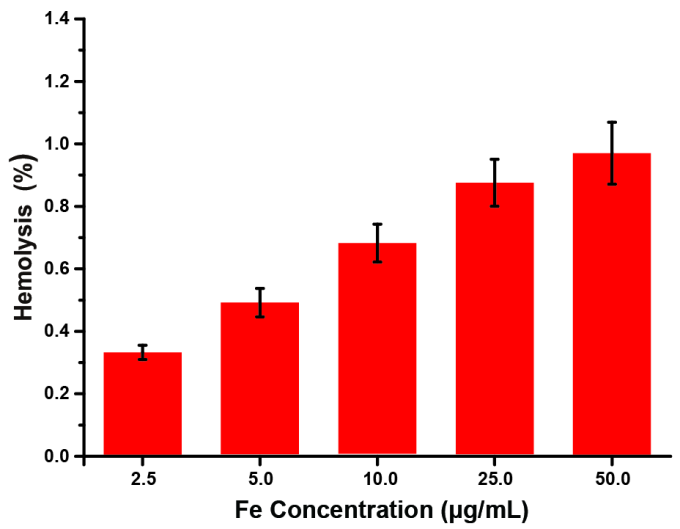


**Figure S11.** *In vitro* hematological analysis of Ab-MZF@DMSA/Gd with various iron concentrations (n = 3).


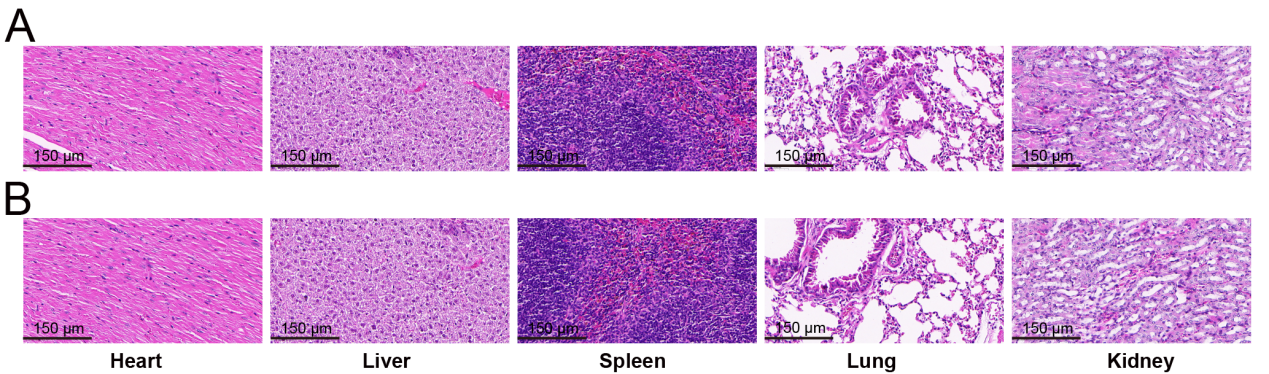


**Figure S12.** H&E stainings of the sections of heart, liver, spleen, lung, and kidney from mice after intravenous injection of A) PBS or B) Ab-MZF@DMSA/Gd.


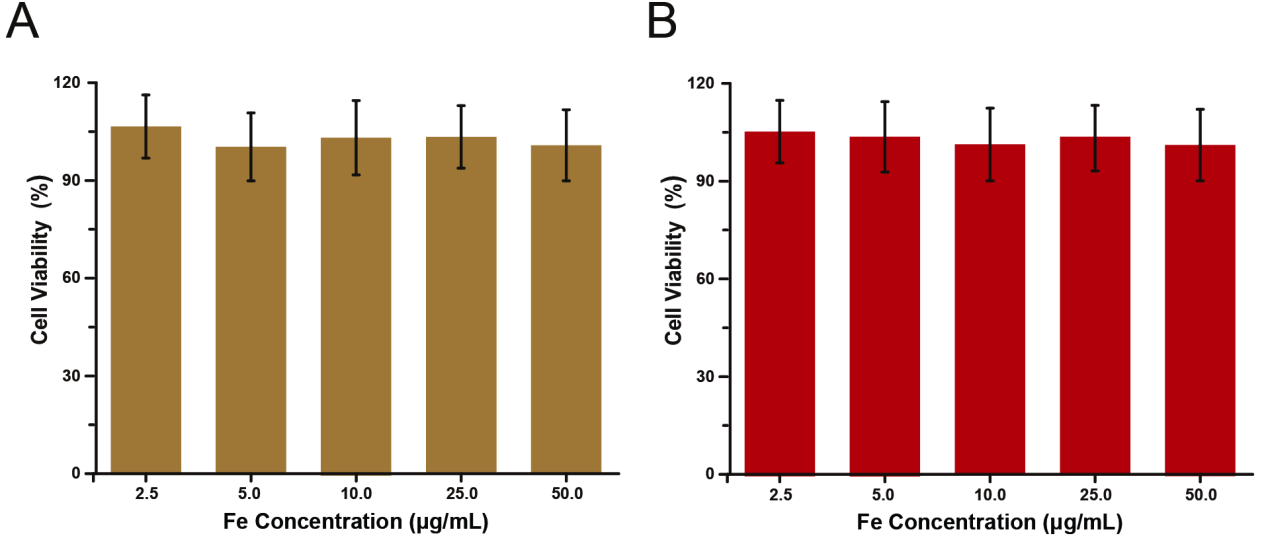


**Figure S13.** Cytotoxicity of Ab-MZF@DMSA/Gd with various iron concentrations on A) SH-SY5Y and B) BCECs (n = 3).


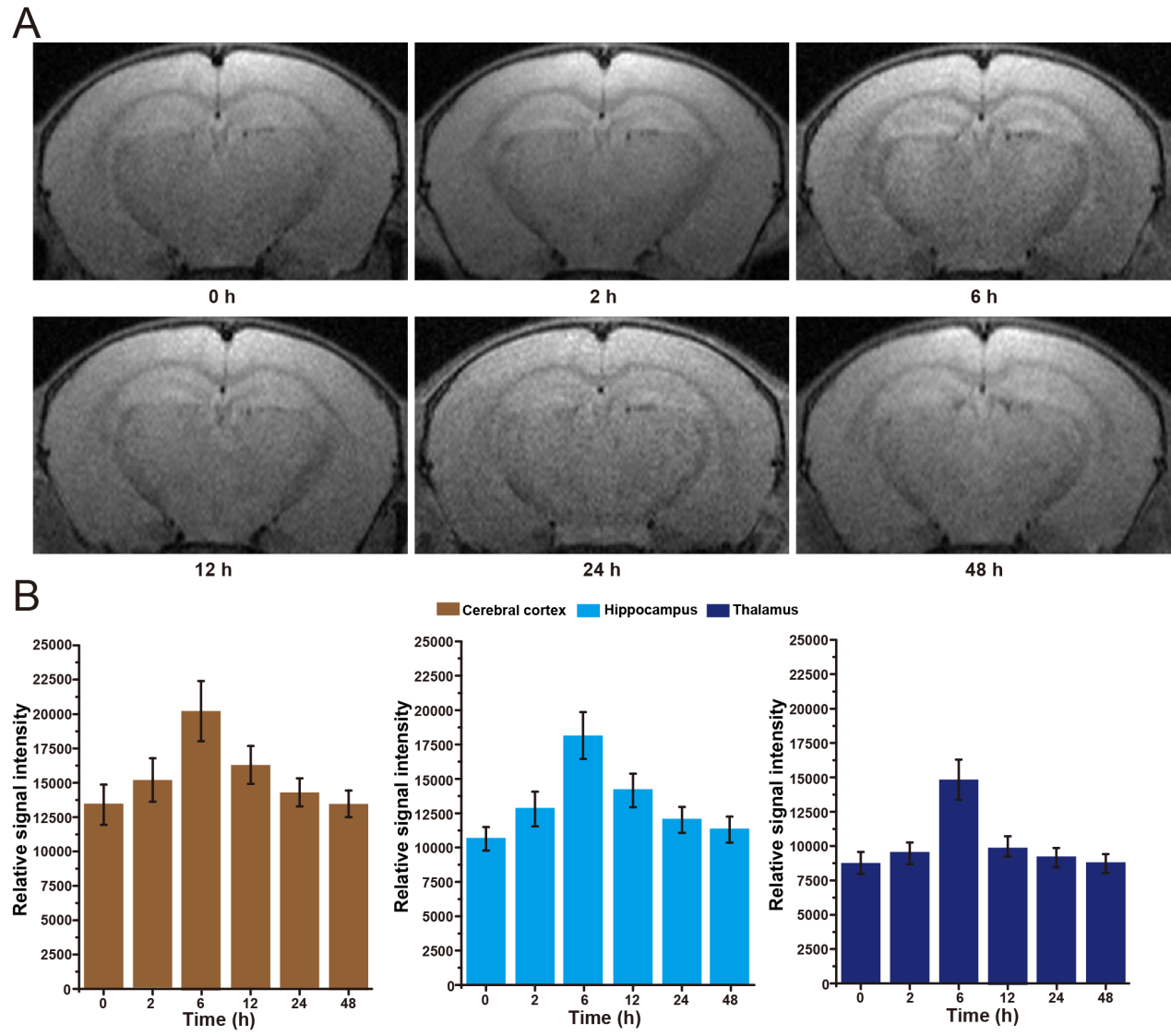


**Figure S14.** A) T1WI images and B) quantification of T1WI signal intensities of 12-week-old AD mice brains at various time points before and after Ab-MZF@DMSA/Gd nanoparticles injection (n = 3).

**
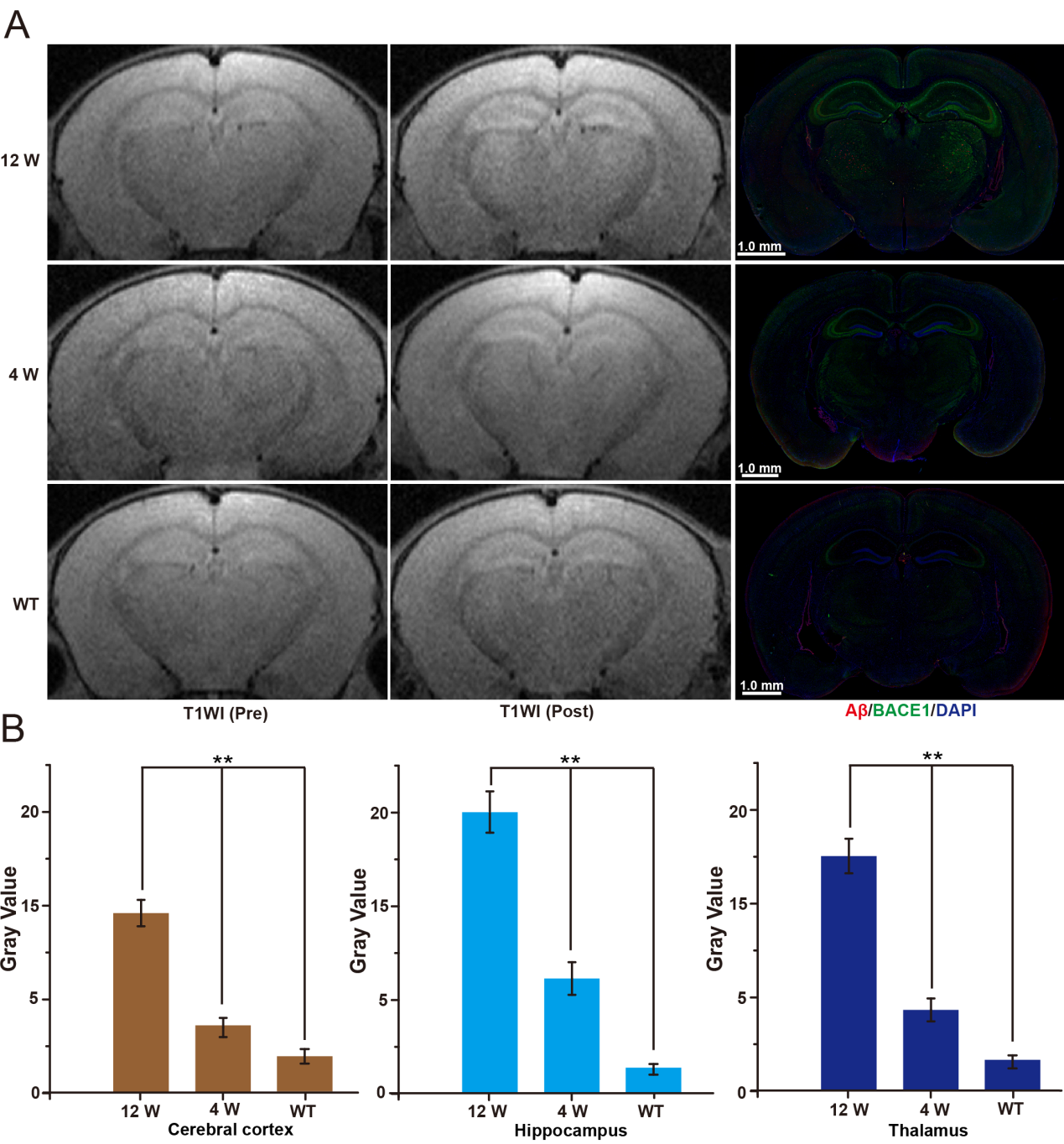
Figure S15.** A) Coronal T1WI images and LSCM images of BACE1-Aβ immunofluorescence of the brains in 12-week-old AD mice, 4-week-old AD mice and WT mice before and 6 hours after Ab-MZF@DMSA/Gd injection (n = 3). B) Comparison of fluorescence gray values of BACE1 of the thalamus, the cerebral cortex and the hippocampus in 12-week-old AD mice, 4-week-old AD mice and WT mice 6 hours after Ab-MZF@DMSA/Gd injection (n = 3). Statistical significance is indicated ( **p < 0.01) by Student’s t-test.


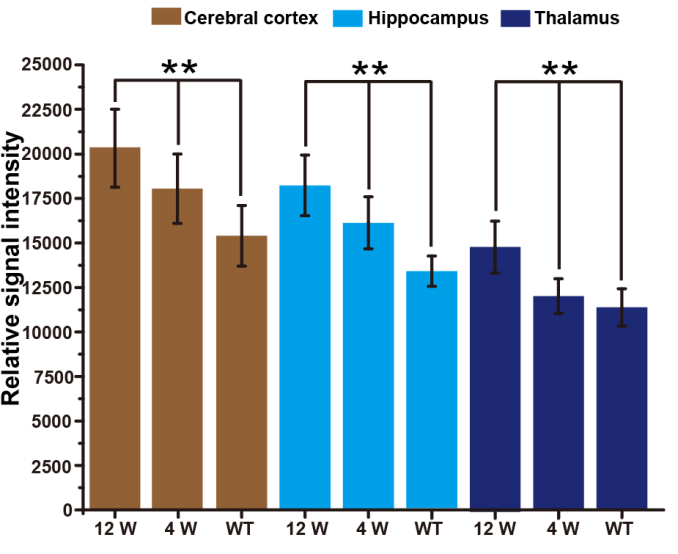


**Figure S16.** Quantification of T1WI signal intensities of the cerebral cortex, the thalamus and the hippocampus in 12-week-old AD mice, 4-week-old AD mice and WT mice 6 hours after Ab-MZF@DMSA/Gd nanoparticles injection. (n = 3). ***p* < 0.01.


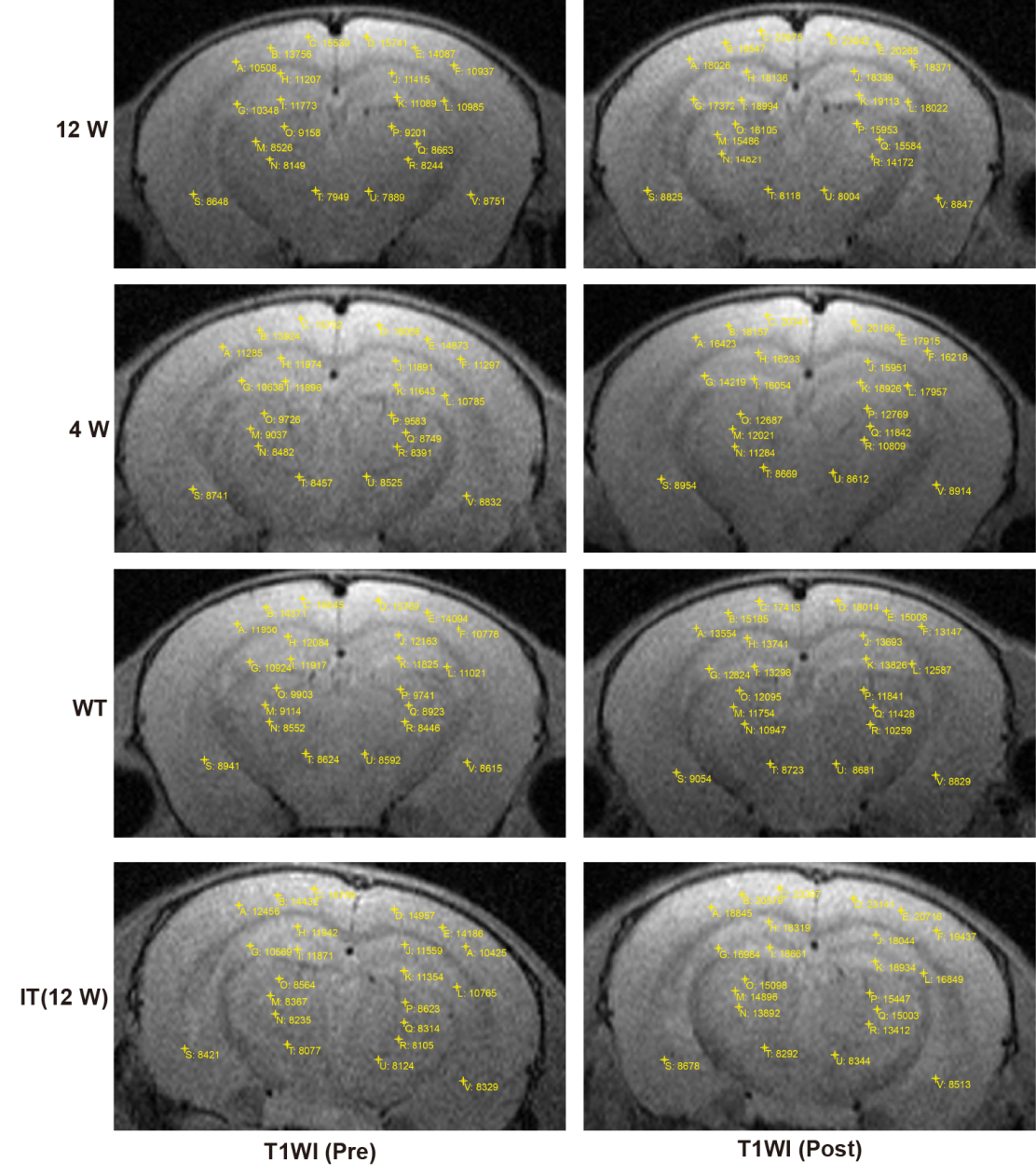


**Figure S17.** The images with annotated T1WI signal intensity values in various brain regions of all groups of mice.


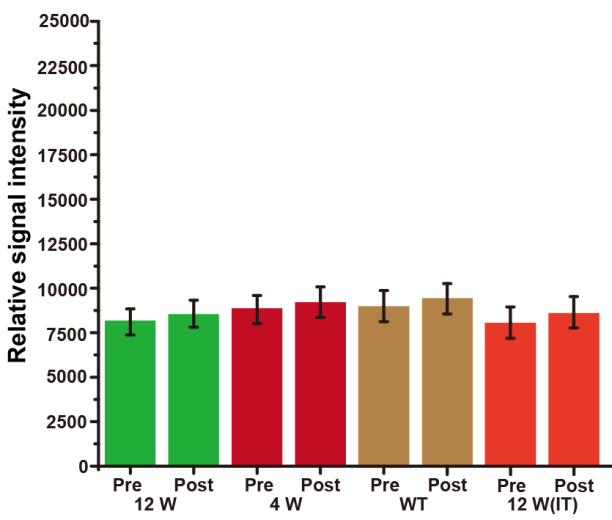


**Figure S18.** Quantification of T1WI signal intensities of other brain regions among all groups before and 6 hours after Ab-MZF@DMSA/Gd injection (n = 3).

**
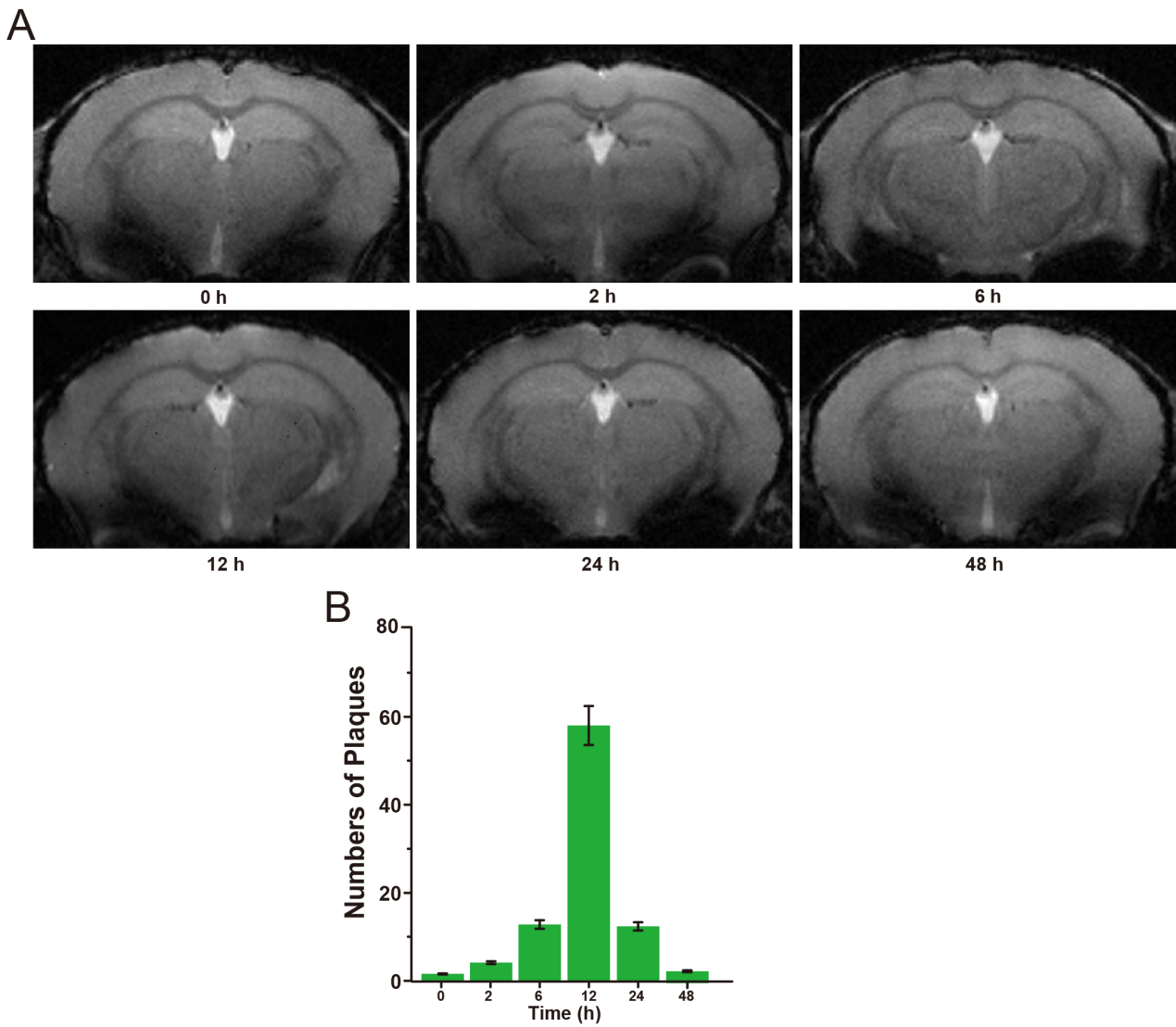
Figure S19.** A) SWI images and B) numbers of plaques based on SWI images of the brains in 12-week-old AD mice before and after Ab-MZF@DMSA/Gd nanoparticles injection. (n = 3).

**
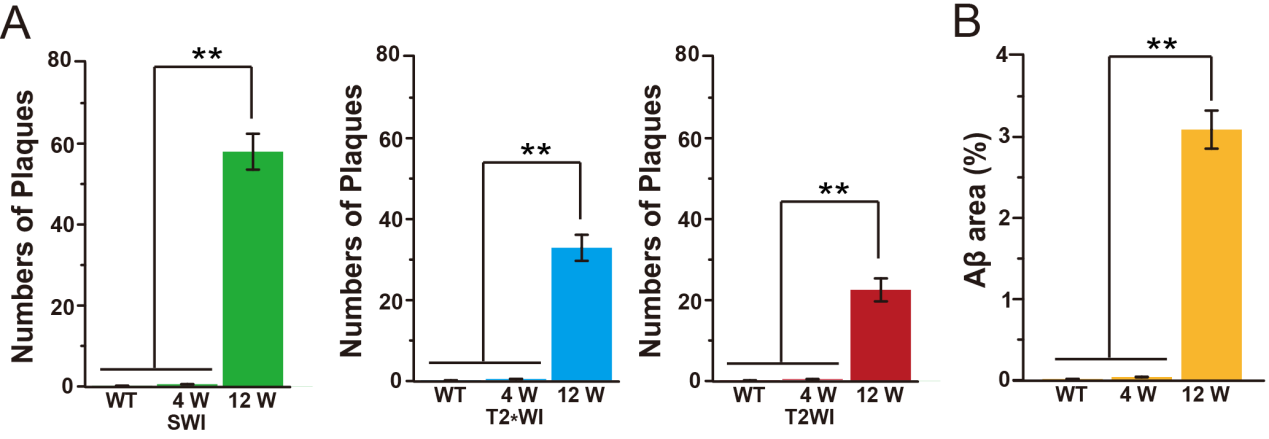
Figure S20.** Quantitative analysis of plaque counts in A) SWI, T2_*_WI and T2WI images of brain sections from WT mice, 4-week-old AD mice, and 12-week-old AD mice 12 hours after Ab-MZF@DMSA/Gd injection (n = 3). B) Quantitative analysis of the area occupied by Aβ plaques in the corresponding brain sections of WT mice, 4-week-old AD mice, and 12-week-old AD mice via immunofluorescent staining (n = 3). Statistical significance is indicated ( **p < 0.01) by Student’s t-test.


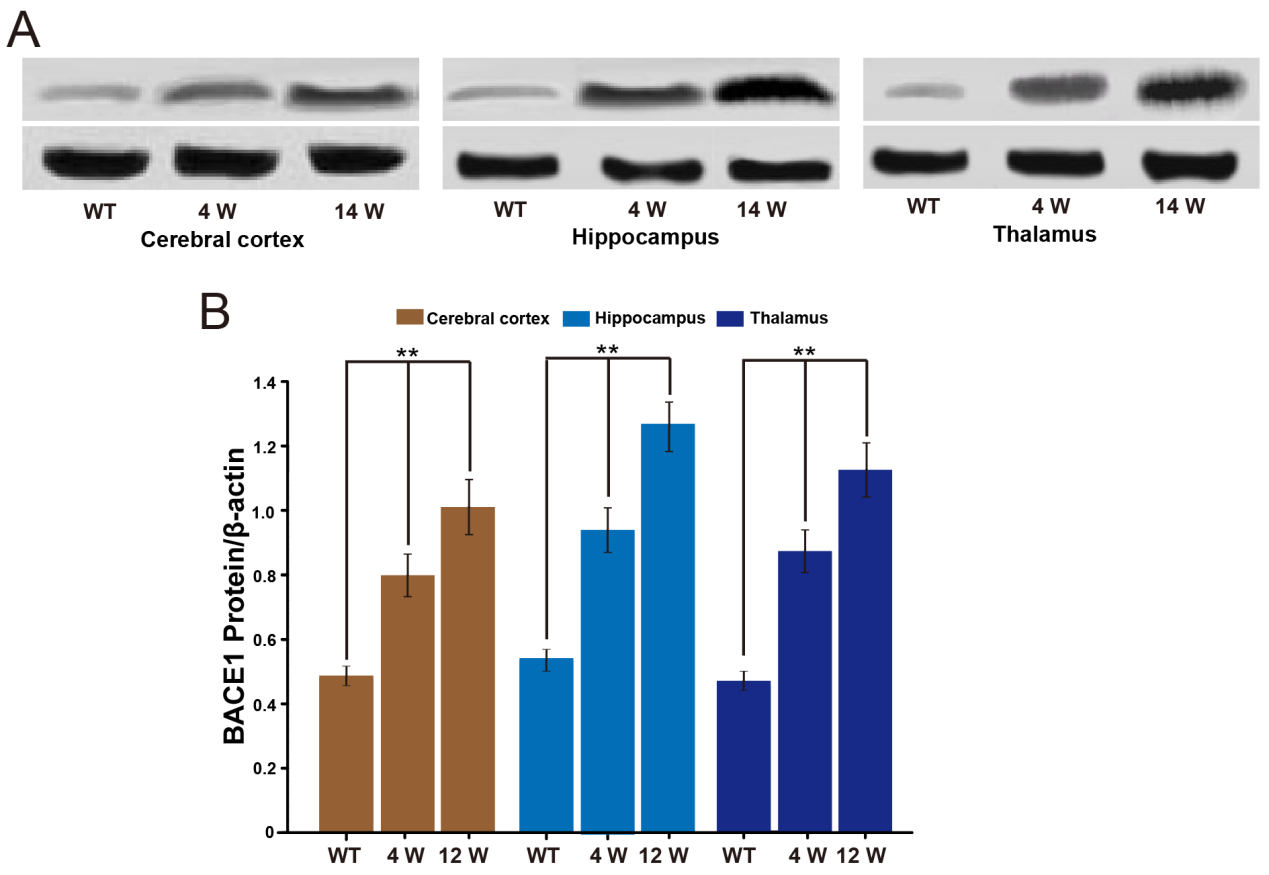


**Figure S21.** A) Relative expressions of BACE1 protein by Western blot. B) Quantification of BACE1 protein expression by Western blot. (n = 3). Statistical significance is indicated ( **p < 0.01) by Student’s t-test.


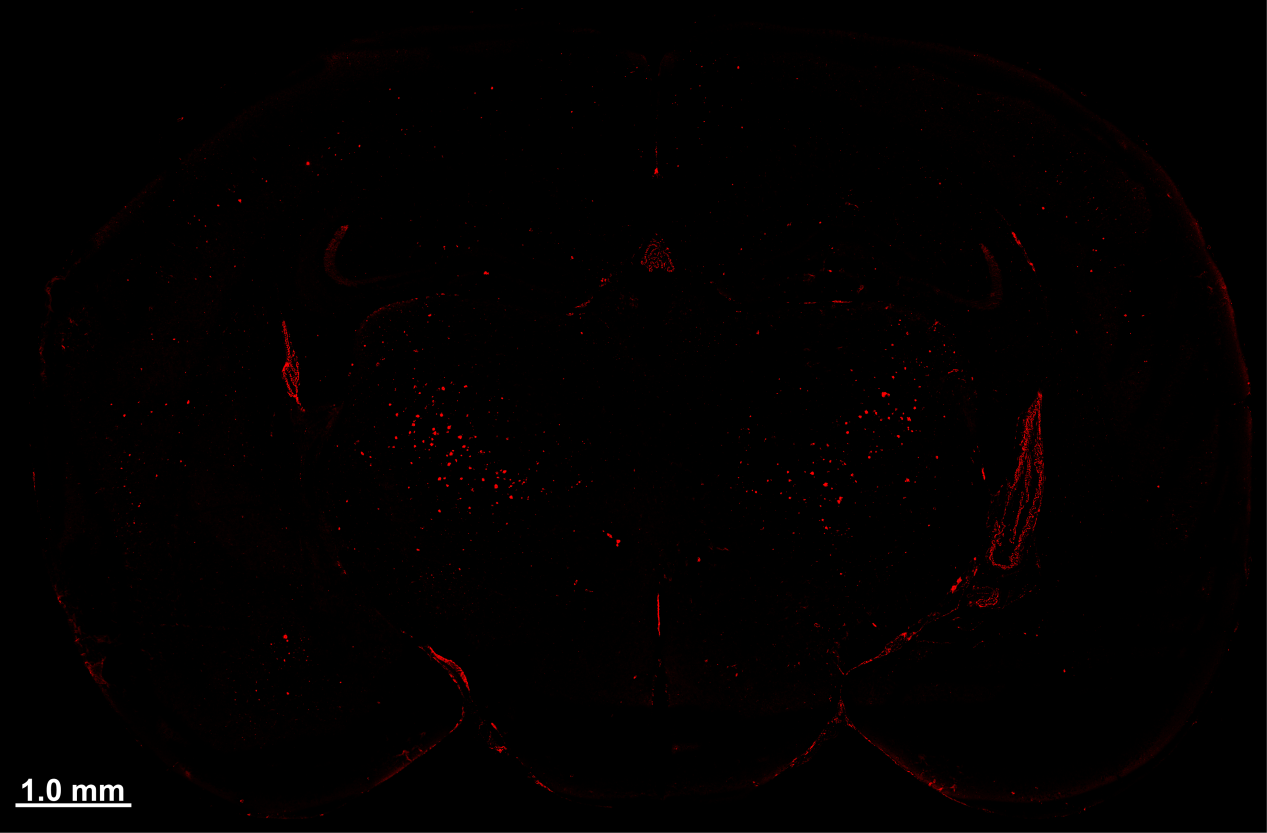


**Figure S22.** LSCM image of Aβ immunofluorescence of the brains in 12-week-old AD mice 6 hours after Ab-MZF@DMSA/Gd injection.

**
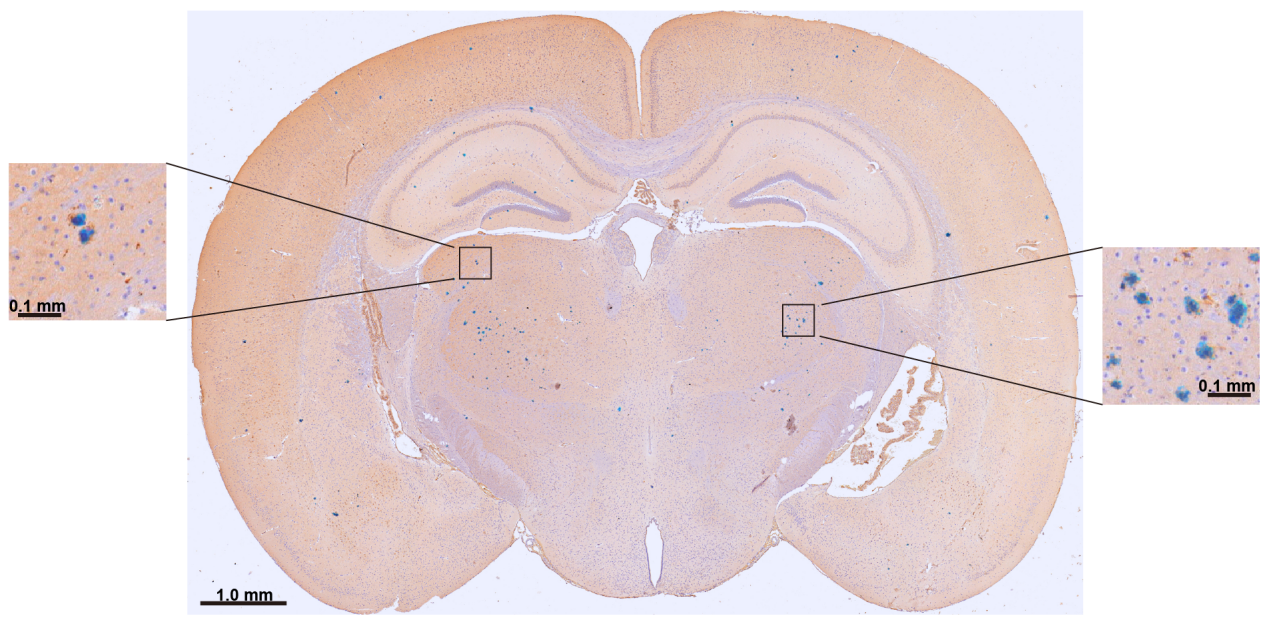
Figure S23.** Coronal brain sections of 12-week- old APP/PS1 mice co-stained for Aβ-Prussian blue. Inset: Magnification of a region displaying co-localization of Aβ plaques (brown) and Prussian blue stained iron oxide (blue).


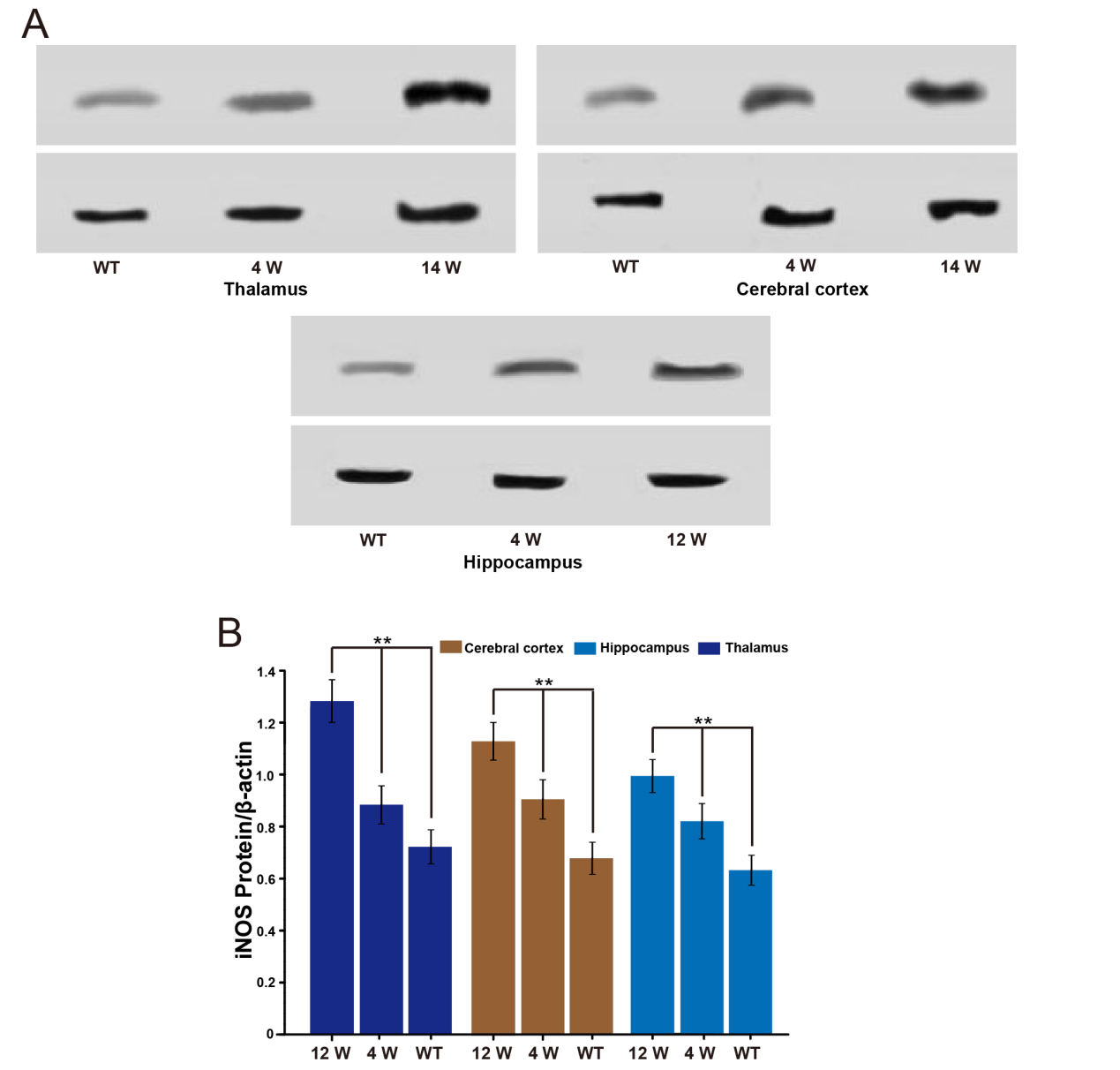


**Figure S24.** A) Relative expressions of iNOS protein by Western blot. B) Quantification of iNOS protein expression by Western blot. (n = 3). Statistical significance is indicated ( **p < 0.01) by Student’s t-test.


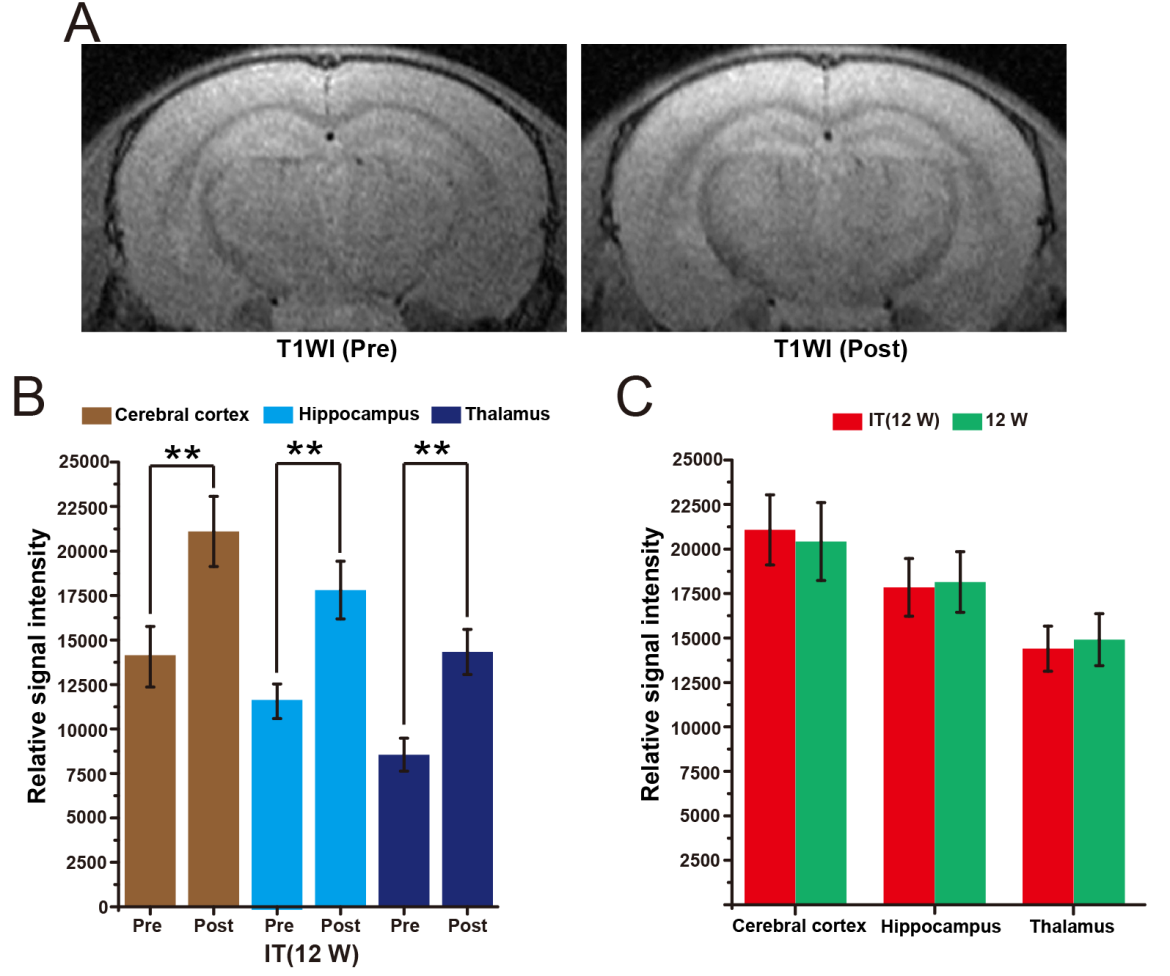


**Figure S25.** A) Coronal T1WI images of the brains in 12-week-old AD mice before and 6 hours after Ab-MZF@DMSA/Gd via intrathecal injection (n = 3). B) Quantification of T1WI signal intensities of the thalamus, the cerebral cortex and the hippocampus in 12-week-old AD mice before and 6 hours after Ab-MZF@DMSA/Gd via intrathecal injection (n = 3). C) Comparison of quantification of T1WI signal intensities of the thalamus, the cerebral cortex and the hippocampus in 12-week-old AD mice and 12-week-old AD mice (IT) 6 hours after Ab-MZF@DMSA/Gd nanoparticles injection (n = 3). Statistical significance is indicated ( **p < 0.01) by Student’s t-test.


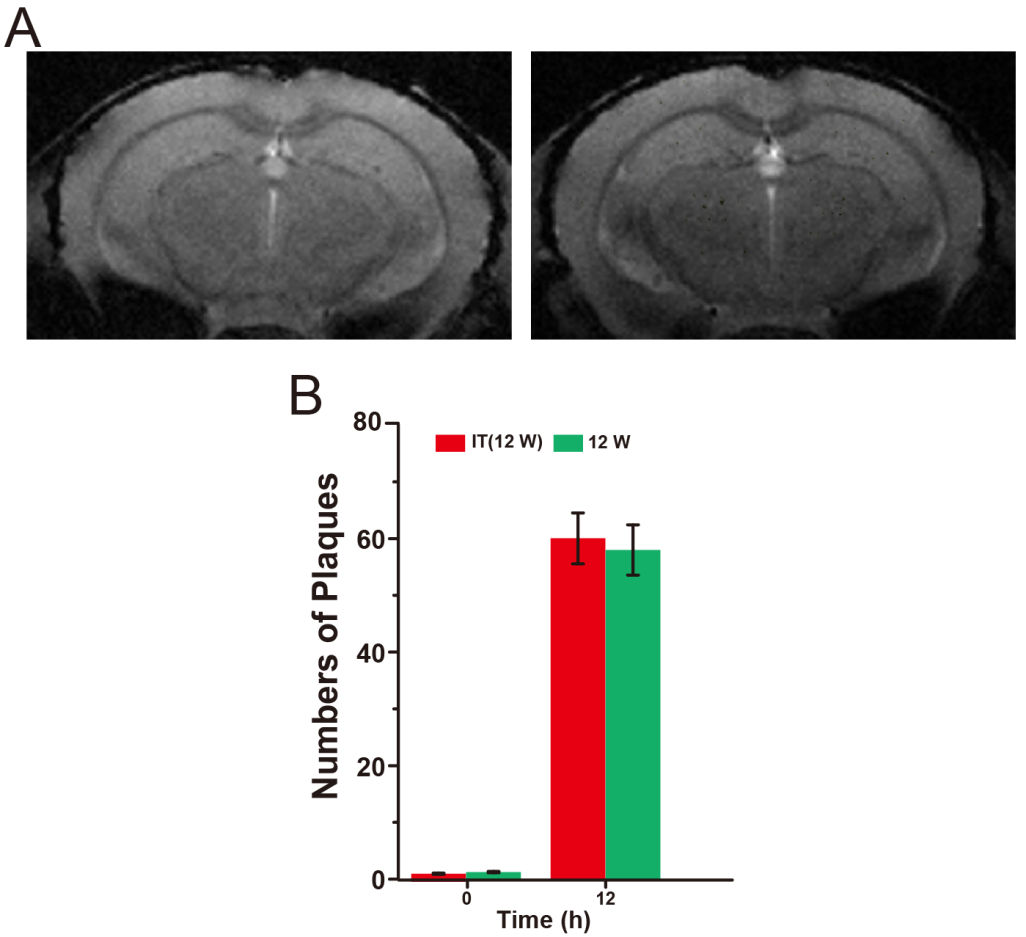


**Figure S26.** A) Coronal SWI images of the brains in 12-week-old AD mice mice before and 12 hours after Ab-MZF@DMSA/Gd via intrathecal injection (n = 3). B) Comparison of numbers of plaques based on SWI images of the brains in 12-week-old AD mice and and 12-week-old AD mice (IT) before and 12 hours after Ab-MZF@DMSA/Gd nanoparticles injection. (n = 3).


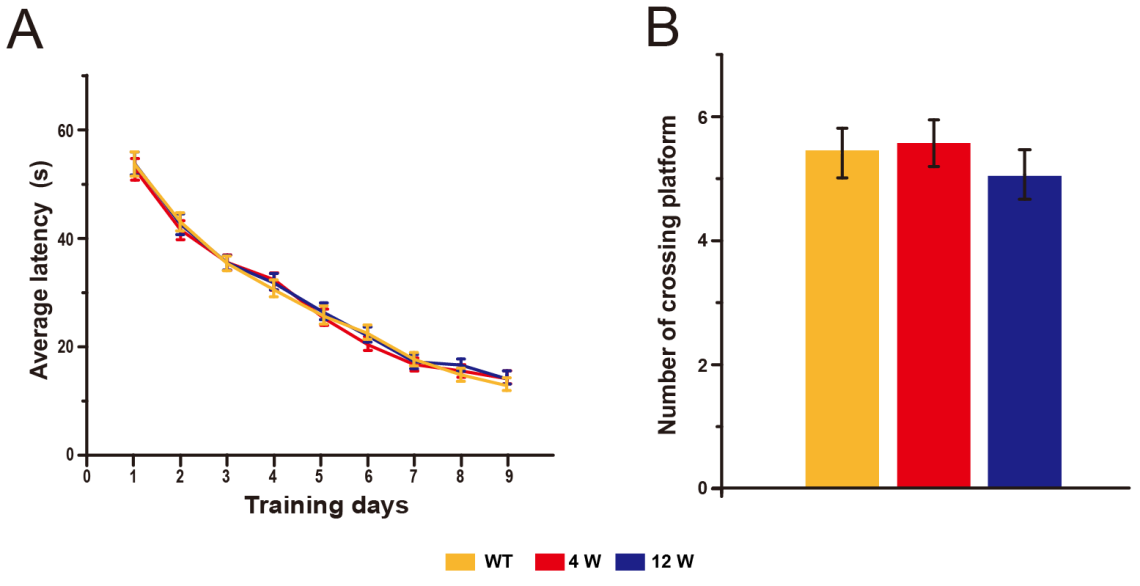


**Figure S27. A)** Comparison of latency in the Morris water maze test across different training sessions for each group of mice. B) Comparison of the number of times each group of mice crossed the target platform in the spatial exploration test.
